# γ-Cyclodextrin-graphene quantum dots-chitosan modified screen-printed electrode for sensing of fluoroquinolones

**DOI:** 10.1007/s00604-023-05646-w

**Published:** 2023-01-19

**Authors:** Manuel Bartolomé, M. Laura Soriano, M. Jesús Villaseñor, Ángel Ríos

**Affiliations:** 1 Department of Analytical Chemistry and Food Technology, Faculty of Chemical Science and Technology, University of Castilla-La Mancha, 13071 Ciudad Real, Spain; 2 Regional Institute for Applied Chemistry Research (IRICA), 13071 Ciudad Real, Spain; 3 Department of Analytical Chemistry, University of Córdoba, Campus of Rabanales, Marie Curie, 14071 Córdoba, Spain; 4 Department of Analytical Chemistry, Industrial Engineering School, University of Castilla-La Mancha, 13071 Ciudad Real, Spain

**Keywords:** Voltammetric approach, Bionanocomposite assembly, Graphenic nanostructure, Animal-source foods, Host–guest receptors

## Abstract

An innovative electrochemical approach based on screen-printed carbon electrodes (SPCEs) modified with graphene quantum dots (GQDs) functionalized with γ-cyclodextrin (γ-CD) and assembled to chitosan (CHI) is designed for the assessment of the total content of fluoroquinolones (FQs) in animal source products. For the design of the bionanocomposite, carboxylated graphene quantum dots synthesized from uric acid as precursor were functionalized with γ-CD using succinic acid as a linker. Physic-chemical and nanostructural characterization of the ensuing nanoparticles was performed by high-resolution transmission scanning microscopy, dynamic light scattering, Z potential measurement, Fourier transformed infrared spectroscopy and X-ray diffraction. Electrochemical properties of assembled bionanocomposite like potential difference, kinetic electronic transfer constant and electroactive area among other parameters were assessed by cyclic voltammetry and differential pulse voltammetry using potassium ferricyanide as redox probe. The oxidation behaviour of four representative quinolones with distinctive structures was studied, obtaining in all cases the same number of involved e^−^ (2) and H^+^ (2) in their oxidation. These results led us to propose a single and consistent oxidation mechanism for all the checked analytes. The γ-CD-GQDs-CHI/SPCE sensor displayed a boosted electroanalytical performance in terms of linear range (4–250 µM), sensibility (LOD = 1.2 µM) and selectivity. This electrochemical strategy allowed the determination of FQs total amount in complex processed food like broths, bouillon cubes and milkshakes at three concentration levels (150, 75 and 37.5 µM) for both equimolar and different ratio FQs mixtures with recovery values ranging from 90 to 106%.

**Graphical abstract:**

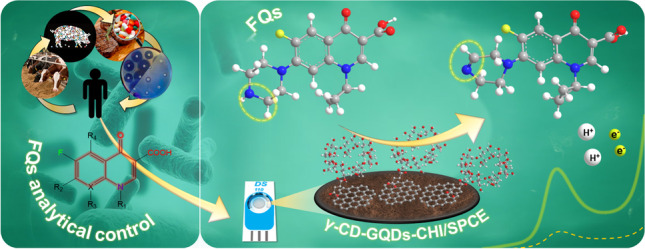

**Supplementary Information:**

The online version contains supplementary material available at 10.1007/s00604-023-05646-w.

## Introduction

Fluoroquinolones (FQs) are a family of synthetic drugs, currently submitted to extensive research, because of their antimicrobial activity against a wide spectrum of Gram-positive and Gram-negative aerobic pathogens. In fact, the third generation of this family of drugs is still under development and clinical studies. Their action mechanism focuses on the inhibition of DNA synthesis by the suppression of bacterial DNA gyrase enzyme activity, producing so the death of bacteria [1]. All FQs have been widely used in both veterinary field and human medicine for the treatment of infection diseases related to respiratory, gastrointestinal, urinary tract, skin and soft tissue, becoming an extremely capital resource owing to their bioavailability, low side effects, simple administration, easy and homogeneous distribution through tissues and fluids, as well as high effectiveness [2].

Since the beginning of current century, FQs have been used in an abusive way by the livestock industry, as well as in poultry and fish farming, leading to the occurrence of quinolone residues in the processed products derived from these sectors. That is the reason why several studies have detected the appearance of quinolone-resistant microorganism due to their unaware and unwitting consumption in recent years [3–6]. This fact involves serious health implications because of the reduced efficacy of FQs-based drugs towards infection and disease treatments in animals and humans; then, European directives have been established regarding the maximal allowed amount of quinolones in food, which currently ranges from 30 to 800 μg/kg depending on the quinolone family member [7, 8].

Thus, there is an urgent need to look for sensitive and realistic methodologies capable of monitoring FQs in diverse scenarios. Most of current analytical techniques involve trained staff and expensive instrumentation such as chromatography [9], capillary electrophoresis [10], fluorescence [11] and Raman spectroscopies [12] and mass spectrometry [13]. Other alternative methods based on simpler instrumentation are the colorimetric/UV–Vis [14] or electrochemical [15] ones, but almost just focused on the detection of one or maybe two FQs species, mainly the same ones (ciprofloxacin or enrofloxacin), which is a great limitation, since actual FQs treatments may involve other several ones. Therefore, so far there is a lack of simple analytical strategies providing determination for a wide number of quinolones while maintaining selectivity against interferences, which is the addressed aim of this research. On the other hand, electroanalytical methodologies offer advantages (low-cost, compact devices ruled by easy and reliable detection principles) displaying promising future trends. Thus, the development of easy, quick and portable devices for qualitative and quantitative controls of global FQs amounts on alimentary matrix suspicious from target residues becomes a great interest topic, due to their inherent associated health issues.

In recent decades, numerous researches have shown the great potential of chemically modified screen-printed electrodes (SPEs) for the detection of antibiotic residues in diverse samples [15–17]. In addition to their many advantages (miniaturization, low cost, quick response time, disposable device), the selectivity and sensibility of these electrochemical devices can be drastically improved by means of simple modifications on the working electrode (WE) surface, which work upgrading the charge transfer rate of redox reactions as well as preconcentrating analytes [10]. Particularly, the incorporation of carbonaceous nanomaterials improves the transduction of electrochemical signals enabling better analytical performances [18].

Graphene quantum dots (GQDs) are one of the newest species of graphene-based nanomaterials, with outstanding optical and electrochemical properties derived from their graphenic structure and electronic quantum confinement, thus standing out their elevated conductivity and great ability to exchange electrons [19]. Additionally, they make up a green and efficient alternative versus other similar carbon-based species because of their non-toxicity, low-cost synthesis, chemical inertness and biocompatibility. The GQDs synthesis procedure usually entails hydrophilic edges and free oxygenated groups (–COOH, –OH) as fitted structures that allow interaction with a large number of organic molecules, enabling so diverse recognition sensing systems, which constitutes a versatile support providing a wide range of functionalization possibilities [20].

Functionalization of nanomaterials with selected cavitands as specific and versatile recognition systems is one of the latest trends for the development of electrochemical sensors in the food and pharmaceutical field. Within this topic, cyclodextrins (CDs), family of cyclic oligosaccharides with variable size (α, β and γ) and with truncated cone shape, stand out because of their ability of forming stable inclusion complexes with specific molecules as targets mainly based on non-covalent interactions. Different authors have already described the formation of inclusion complexes with specific recognition between CDs and FQs [21–23] based on host–guest interactions and hydrogen bonding. In addition, GQDs can be easily functionalized with CDs through interactions of diverse nature [24, 25] constituting thus a selective, versatile and efficient recognition support. But the use of CD-GQDs assemblies has been scarcely checked for electrochemical detections, being still a field to deep in with a scope plenty of chances. So, electroanalytical methods based on inclusion complexes as recognition strategy to afford selectivity becomes a promising future strategy. Besides in our specific research, a multianalyte sensing, CDs exhibit an outstanding advantage since they offer a steric interaction (size based and non-highly specific), which enables both a global FQs determination or just for an individual compound.

With the aim to enhance both durability and robustness of the designed nanocomposite, chitosan (CHI) was added. CHI is a natural cationic polymer containing amino and hydroxyl groups and widely employed as anchoring tool for electrode modifications due to its mechanical strength, chemical stability, good adsorption, as well as homogenous and thinner film layering deposition on electrode surface [16]. Positively charged amino groups of CHI can interact with carboxyl ones of GQDs via electrostatic interactions, anchoring the modification at WE surface and avoiding so the solubilization of the GQDs during the subsequent washing steps. Since CHI does not exhibit electrical conductivity, it is generally combined with conducting materials to enhance its performance for electrochemical sensing applications.

In this way, we proposed an innovative, miniaturized and portable electroanalytical tool for the control of global FQs amount in complex food processed products that means realistic scenarios where their determination should be considered. The sensing platform consists of a screen-printed carbon electrode (SPCE) modified with a novel bionanocomposite based on gamma-cyclodextrin functionalized graphene quantum dots supported on a chitosan membrane (γ-GQDs-GQD-CHI) to increase the electroactive area and preconcentrate FQs onto the electrode. The design of the finally selected γ-CD-GQD-CHI composite was accomplished through extensive synthesis and characterization researches, namely, nanostructural, physic-chemical and electrochemical studies. This pioneering bionanocomposite assembly has provided great advantages in terms of sensibility (GQDs), reusability (chitosan) and selectivity (γ-CD) for the global estimation of FQs mixtures in assorted composition and ratios, what so far was not available yet through any other research. Besides, this research provides for the first time a single oxidation mechanism for the whole FQs family based on the found electrochemical insights.

Finally, current challenges and trends inside this topic should explore the incorporation of CDs as versatile recognition tools within novel engineered electrochemical bio/composites, besides the nanostructuration implementation as a way to enhance electroactive areas associated to the increase of surface area/volume ratios. To reach this effect, it can be also consider the inclusion of safe biomaterials such as GQDs to provide improvement of electron transfer rate and chitosan to provide strength, chemical stability and homogeneity in an effort to boost innovative miniaturized electrochemical methodologies for the resolution of realistic problems of current importance inside the alimentary/environmental fields.

## Experimental

### Reagents and solutions

All solutions were prepared with analytical grade reagents without previous purification and using deionized water (18.2 MΩ·cm resistivity) provided by a Milli-Q system (Millipore, Bedford, MA, USA).

Enrofloxacin (Enro, ≥ 98%), lomefloxacin (Lome, ≥ 98%), norfloxacin (Nor, ≥ 98%), danofloxacin (Dano, ≥ 98%), ciprofloxacin (Cip, ≥ 98%), ofloxacin (Oflo, ≥ 98%), α-, β- and γ-cyclodextrin (> 98%), succinic acid (> 99.5%), chitosan (low molecular weight), sodium hydroxide (> 98%), sodium chloride (> 99%), potassium chloride (> 99%), magnesium sulphate (> 99%), potassium ferricyanide (> 98%), sodium acetate (≥ 99%), sulphuric acid (≥ 95–97%), sodium acetate (≥ 99%), *N*,*N′*-diisopropylcarbodiimide (≥ 98.0%), L-alanine (> 98%), L-lysine (> 98%), L-glutamic acid (> 98%), L-phenylalanine (> 98%) and amylose type III from potato starch were purchased from Sigma Aldrich (St. Louis, MO). Riboflavin (Rib, > 98%), L-ascorbic acid (99.7%), acetone (0.01% water content), acetic acid (99.7%) and hydrochloric acid (37%) were bought from Panreac (Barcelona, Spain). Amylopectin from potato starch were obtained from Fluka (Steinheim, Germany). Uric acid was purchased from Alfa Aesar (Ward Hill, MA).

Stock solution of potassium ferricyanide (5 mM) was prepared in KCl (0.1 M). CHI stock solution 0.5% (w/w) was first dissolved in 1% acetic acid and sonicated until complete dissolution, adjusting then to pH = 5 with NaOH 1 M. FQs stock solutions (1.5 mM) were dissolved in acetate buffer (0.1 M, pH = 5), except for norfloxacin, which was first prepared in 5% acetic acid and subsequently adjusted to pH = 5. All working interference solutions were prepared in deionized water.

### Instrumentation

Electrochemical measurements were performed with a CHI842D electrochemical analyser (CH Instruments; TX, USA). Different screen-printed electrodes with unmodified carbon (DRP-110) and diverse nanomaterials as WEs, such as multi-walled carbon nanotubes (MWCNTs, DRP-110 CNT), single-walled carbon nanotubes (SWCNTs, DRP-110 SWCNT), gold nanoparticles (AuNPs, DRP-110 GNP) and graphitized carbon nanofibers (NFCs, DRP-C110 CNF), were purchased from Dropsens (Oviedo, Spain). A DRP-BICAC70311 connector was used as interface between the potentiostat unit and the SPEs (Dropsens).

Size, morphology and *d* spacing (spacing between sheets) of γ-CD-GQDs were examined by transmission electron microscopy (TEM) using a JEOL JEM 1400 and high-resolution (HR) JEOL 2100 models. For sample preparation, a drop of γ-CD-GQDs (2 g L^−1^) solution was deposited in a TEM grid, and subsequently dried at room temperature for further analyses by HR-TEM.

Fluorescence emission and excitation spectra of the diverse GQDs were taken by a Photon Technology International QuantaMasterTM spectrofluorometer with a 10-mm quartz cuvette at room temperature. Emission and excitation slit widths were set at 1 nm. An interface FelixGX software was used to collect and process all fluorescence data. UV–visible spectra were performed with a SECOMAN-UVI Light (XS.2) spectrophotometer equipped with a LabPower V3 50 and 10-mm quartz cuvettes. Infrared spectra (IR) of GQDs were performed in a Shimadzu infrared instrument (IR-Affinity-1S model and DTGS Standard detector) equipped with an attenuated total reflectance crystal (ATR). The crystal puck was of ZnSe. X-ray diffraction spectra were recorded by a Philips Panalytical X-ray diffractometer (Malvern, UK), Model X’Pert MDP with Cu Ka 1 radiation, automatic divergence slit, graphite monochromator and proportional xenon gas sealed detector. A thick layer of the nanomaterial was obtained by subsequent 20 µL γ-CD-GQDs drop-castings (up to 5 mL as total volume of the 4.1 g L^−1^ nanodot solution) and dried under an IR lamp inside a cavity of a homemade transparent Si/SiO_2_ sample holder.

Particle size distribution and zeta potential measurements of carboxylated GQDs, α-, β- and γ-CD-GQDs were performed by dynamic light scattering (DLS) using a Zetasizer Nano Series DLS ZEN3500 (Malvern Instruments Limited, Spain).

A rotavapor (Buchi model R-300, UK), an analytical balance (Mettler AE 240), a Selecta ultrasound bath, a Crison pH meter with a combined glass electrode, a Minicen high-speed mini centrifuge (OrtoAlresa, Spain) and a high-speed centrifuge with refrigeration (centrofriger-BL-II model 7001669, JP Selecta, Spain) were also used.

### Sample treatments

Commercial samples of chicken bouillon, veal bouillon cubes, chicken broth, veal broth and strawberry milkshake were acquired in local supermarkets. The samples here studied are complex matrix featured, mainly made up of oils and fats (for broths) and of proteins and fats (for dairy products). However, they also contain other excipients in smaller proportions, such as vitamins, electrolytes, extracts of animal and vegetable origin and different additives. The detailed composition of these nutritional supplements is below reported.

- Chicken bouillon: sodium chloride, starch, yeast extract, chicken extract, onion extract, oleic acid, flavour enhancers (glutamate and sodium ribonucleotides), some flavourings and spices.
- Veal bouillon: sodium chloride, yeast extract, veal extract, onion and carrot extract, palm fat of vegetable origin, caramel dye, flavour enhancer (glutamate) and natural antioxidants (rosemary extract).
- Chicken broth (without colouring and preservatives): chicken (2%), corn modified starch, sodium chloride, different vegetables (0.1%), oleic acid and some spices.
- Veal broth: veal meat (3.5%), veal extract (0.05%), different vegetables (0.8%), sodium chloride, natural flavourings.
- Milkshake strawberry flavour: milk (0.6% fat content), glucose, stabilizers (sodium triphosphates, microcrystalline cellulose and carboxymethylcellulose), emulsifiers (fatty acids monoglycerides and diglycerides), alimentary dye (cochineal carmine) and flavouring.
- The developed electrochemical approach was applied to above-described samples, once submitted to the sampling treatment next described.
- Bouillon cubes: 2 g of the tablet was dissolved in 75 mL of 0.1 M acetate buffer (pH 5), then, the sample was spiked with appropriate amounts of FQs to reach the stablished concentration levels (150, 75 and 37.5 µM) and diluted up to 100 mL with the buffer. Later, 6 mL of each sample was twice centrifuged (14,000 rpm; 15 min), leading to the appearance of three well-defined phases. Intermediate serum phase was then isolated in the Eppendorf from the lipidic phase (top) and from the precipitated one (bottom) between centrifugations. Finally, samples were diluted to their corresponding level with acetate buffer.
- Broth samples: 2.5 mL of broth was spiked with appropriate FQs amounts to reach the stablished concentration levels and subsequently set up to 5 mL with 0.1 M acetate buffer pH = 5. Then, these samples were twice centrifugated, isolating the serum from other phases and finally diluting to their corresponding level with 0.1 M acetate buffer.
- Milkshake: 12.5 mL of milkshake was spiked with suitable FQs amount to reach the stablished concentration levels. To denature milk proteins for avoiding their interference in the electrochemical measurements, the sample solution was heated at 40 °C with 520 µL of acetic acid 10% (v/v) for 5 min under soft stirring conditions until a cloudiness began to appear. Then, 1.58 mL of 1 M sodium acetate was added to get 0.1 M acetate buffer and this resulting solution was taken to 25 mL as final volume. Sample was now centrifuged twice to separate the serum from precipitated proteins and the pH of the isolated serum was adjusted to 5 with NaOH (0.1 M). Finally, these solutions were diluted to their corresponding level with 0.1 M acetate buffer.

### Fabrication of γ-CD-GQDs-CHI modified SPCE

Fabrication of γ-CD-GQDs-CHI modified SPCEs was accomplished in two steps. Firstly, with the aim of improving electrical properties of electrodes, of removing ink contaminants too, and of achieving a consistent and reproducible analytical response, a surface pretreatment step was carried out. This step was performed by means of anodic oxidation of the WE surface with immersion in 5 mM H_2_SO_4_ solution while applying six CV (cyclic voltammetry) cycles between − 1.2 and 1.2 V with 100 mV s^−1^ as scan rate and a potential step of 2 mV [26]. Secondly, for WE surface modification, solution containing γ-CD-GQDs (3.69 g L^−1^) and CHI 0.05% (w/v) were prepared by mixing appropriate amounts of both components and vortexing for 20 s. Then, a 10 µL aliquot of this solution was drop-casted over WE surface and dried under an infrared lamp. Finally, the modified electrode called as γ-CD-GQDs-CHI/SPCE was rinsed with deionized water before use.

For fabrication of other modified WE electrodes (c-GQDs, α-CD-GQDs and β-CD-GQDs), the experimental procedure was almost the same but using 10 µL drop-casted volumes of the c-GQDs, α-CD-GQDs and β-CD-GQDs solutions previously synthesized (section S1 of Supplementary Material (SM) file).

### Electrochemical procedure

Electrochemical characterization of bare and those modified WEs (c-GQDs; γ-CD-GQDs and γ-CD-GQDs-CHI), which were sequentially evaluated and selected as optimal components for the SPCE sensing device along this research, was performed through CV (cyclic voltammetry) using potassium ferricyanide as redox probe. Thirty microlitres of 5 mM K_3_Fe(CN)_6_ in 0.1 M KCl were used to cover all the three-electrode system (WE, AE, RE), and cyclic voltammograms were registered between − 1 and + 1 V, with scan rates ranging from 10 to 500 mV s^−1^. Electroanalytical FQs studies were carried out by DPV (differential pulse voltammetry), where aliquots of suitable concentrations were drop-casted over modified γ-CD-GCDs-CHI/SPCEs recording the corresponding voltammograms. DPV instrumental conditions were as follows: potential change 4 mV, amplitude 25 mV, pulse width 0.05 s, sampling width 0.0167 s, pulse period 0.5 s and quiet time 2 s. The selected drop volume sample was 30 µL for all experiments and a new drop was used for each measurement, rinsing the electrode surface with deionized water between them.

With regard to the reusability issue of the designed bionanocomposite as WE, after every two measurements, renewal of the electrode surface was reached by immersing the electrode in 5 mM H_2_SO_4_ while scanning six CV cycles between − 1.2 and 1.2 V at 100 mV s^−1^ as scan rate. This anodic oxidation process can be repeated until five times, extending thus the useful life of the modified SPCE up to 10 measurements.

Information relative to “Synthesis of c-GQDs, α-CD-GQDs, β-CD-GQDs, γ-CD-GQDs and γ-CD-GCDs/CHI modifiers” and “Theoretical background parameters for electrochemical characterization” is in detail given in sections S1 and S2 of SM file, respectively.

## Results and discussion

Structural, morphological, and optical characterizations of c-GQDs, α-CD-GQDs, β-CD-GQDs and γ-CD-GQDs are described in detail in section S3 of SM file, also including corresponding Figs. S1-S4 and Table S1.

### Design of electrochemical sensing bionanocomposite

To select the best conditions for sensor performance, experiments were carried out by addressing: (a) different commercial and home-made modified screen-printed electrodes, (b) nature and concentration of different cavitands as modifiers (α-CD-GQDs, β-CD-GQDs and γ-CD-GQDs) based on the host:guest interactions, (c) incorporation of CHI as cationic polymeric supporting membrane and (d) achievement of an activation process to recover modified electrode surface and thus to get reproducibility between measurements. These experiments were performed by DPV for a 1.5 mM enrofloxacin solution in 0.1 M acetate buffer (pH = 5). Enrofloxacin was selected as model analyte for FQs family, since it just contains the characteristic chemical group of these chemical targets, 1,4-dihidroquinoleine ring, displaying so a representative behaviour for all of them.

**Fig. 1:**
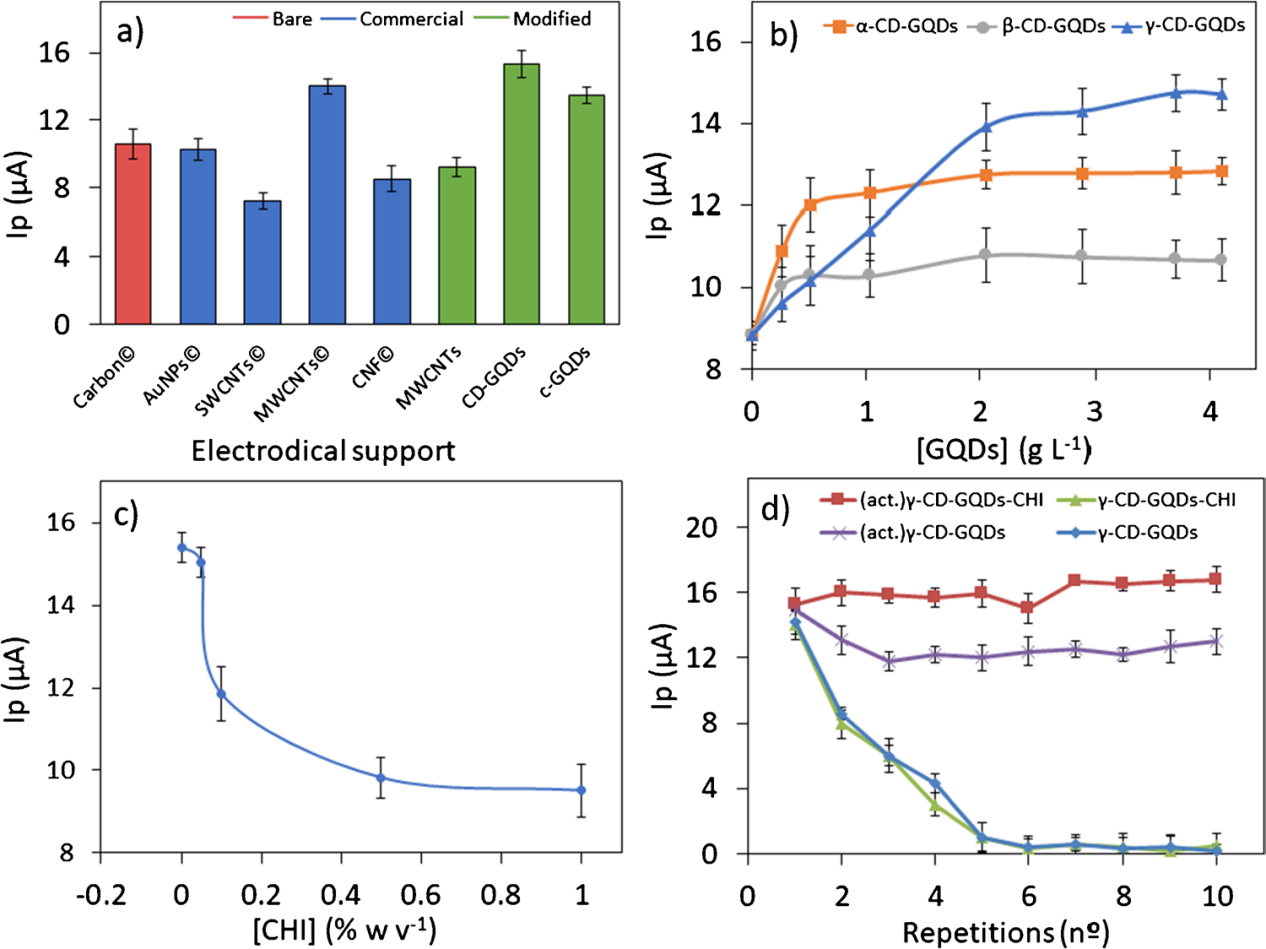
Design of the electrochemical bionanocomposite for the sensing of enrofloxacin 1.51 mM: **a** oxidation peak current obtained for different commercial and home-made screen-printed electrodes. **b** Influence of nature and amount of CD (α-, β- and γ-CD) over sensor performance. **c** Selection of CHI amount. **d** Influence of selected activation/surface renewal procedure. The uncertainty of the results was determined for (*n* = 3) measurements in all cases

Firstly, the influence of different electrodical supports on the sensitivity of enrofloxacin oxidation process was considered. In this study, bare carbon electrodes, commercial modified ones (MWCNTs, SWCNTs, AuNPs and CNFs SPCEs) and home-made modified ones (MWCNTs, c-GQDs and γ-CD-GQDs) were compared onto SPCEs. Due to the high cost, low versatility and lack of novelty of some of these commercially modified electrodes, different SPCEs were also modified employing graphene-based nanomaterials: MWCNTs (0.04 g L^−1^), c-GQDs (9.60 g L^−1^) and γ-CD-GQDs (3.70 g L^−1^), to check their influence over the enrofloxacin oxidation process. As Fig. 1a displays, the best response was achieved for γ-CD-GQDs/SPCE platform, which showed even higher sensibility than the commercially modified ones, as well as evidenced the best suitability of graphene-based species regarding oxidation kinetics, improving thus the sensitivity and sensor performance; so, γ-CD-GQDs modified SPCE was selected in these early stages attending its best *Ip* results.

Regarding behaviour of host–guest systems as a function of their cavity for guest accommodation, influence of different CD sizes was also examined. SPCEs were modified with α-, β- and γ-CD-GQDs at different concentrations ranging from 0.26 to 4.10 g L^−1^ and the corresponding results (*Ip*) are displayed in Fig. 1b, where the highest sensitivity was achieved by WE modification with 3.7 g L^−1^ γ-CD-GQDs solution. These results also indicate the better suitability of the larger CD for FQs electrochemical detection, likely because of suitable thermodynamic interactions (between guest, CD and solvent) and due to the inclusion complexation too (ruled by hydrogen bonding and hydrophobic interactions). Upon this hypothesis, the intramolecular H-bonds formed (likely six) of the β-CDs completed secondary belt give a lower binding-availability to this cavitand, while this H-bond belt is incomplete for the α-CD because of distortion of one glucopyranose unit (that means just four H-bonds). Thus, due to a larger and more flexible structure of γ-CD, a smaller number of intramolecular H-bonds are formed thus favouring the intermolecular binding with FQs, which becomes a crucial point, since favourable net energetic driving forces pull the FQ into the γ-CD cavity [27].

Then, two main drawbacks related to reusability of sensor were addressed, first, the loss of modified layer between measurements due to the high water-solubility of GQDs and, second, the loss of current due to fouling of WE itself.

To avoid the first drawback, a polymeric cationic membrane of CHI was incorporated for anchoring γ-CD-GQDs onto WE surface. At working pH = 5, their charged amino groups may electrostatically interact with the free carboxyl groups of the γ-CD-GQDs, favouring their retention on the polymeric surface. Since CHI is a non-conductive material, its excess could worsen the sensor sensitivity by decreasing the electrode-substrate charge transfer. Thus, a minimal CHI amount enabling the anchoring of GQDs to electrode surface should be taken. Figure 1b shows the *Ip* values for modified electrodes in the absence and presence of CHI within a range between 0.05 and 1.00% (w/v). A 0.05% of CHI was firstly selected as a compromise choice to avoid conductive decrease and signal variation (less than 3.5%) issues.

Finally, the influence of the described activation procedure (“Fabrication of γ-CD-GQDs-CHI modified SPCE” section) to recover the bionanocomposite electrode surface was evaluated. A 1.51 mM enrofloxacin solution was monitored during 10 consecutive analyses (applying six consecutive CV cycles in 5 mM H_2_SO_4_ between every two measurements) and the recorded *Ip* values were compared with those found without any activation procedure. As shown in Fig. 1d, the activation protocol provides a constant and reproducible signal even after 10 measurements (red line), while the analytical signal half decreased after the first analysis when no WE activation surface is performed (green line).

The same experiments were performed in CHI absence to evaluate if the firstly selected chitosan amount enables anchoring the γ-CD-GQDs to the WE surface. As Fig. 1d displays, when activation procedure was performed in CHI absence (purple line), peak intensities decreased after first analysis, due to the loss of electrode modification; thus, it proved the suitable performance achieved by the chitosan modification for at least ten analyses.

With regard to stability considerations of the selected bionanocomposite assembly, although the common experimental procedure for the screen-printed electrode modifications was to prepare them for an almost immediate use, sometimes a period about 3 or 4 days (for instance with a weekend interval) elapsed in many of the achieved tests throughout the evaluation of the analytical performance characteristics or even for the analysis of commercial samples. Therefore, we can assess that sensor once prepared exhibits at least such stability period.

Additionally, the stability of the sensing assembly (γ-CD-GQDs/SPCE) was studied over a 5-day period by means of DPV enrofloxacin scans at different time intervals. This study evidenced satisfactory results since both current and potential oxidation peak remained unchanged with a relative standard deviation lower than 5%.

### Electrochemical characterization of selected γ-GQDs-CHI modified SPCE

Relevant electrochemical parameters of the sensing bionanocomposite (*E*^0′^, *k*^0^, *A*, *Cdl*) were evaluated by CV along its different synthesis and engineering stages in an effort to improve its electrochemical behaviour. With this aim, voltammograms with scan rates ranging from 10 to 500 mV of the bare electrode and of the different nanomaterials steadily used as selected modifiers (c-GQDs, γ-CD-GQDs and γ-CD-GQDs-CHI, respectively) were recorded using 5 mM potassium ferricyanide in 0.1 M KCl and displayed at Fig. S5. Theoretical background to support the electrochemical parameter calculations is detailed in S2 section (SM) and the obtained results along this electrochemical characterization are shown in Table 1.

**Table 1:** Electrochemical characteristic parameters for bare, c-GQDs, γ-CD-GQDs and γ-CD-GQDs-CHI modified SPCEs

Electrodes	*ΔE* range (V)	*k*^0^ (cm s^−1^)	*A* (cm^2^)	*Cdl* (µF cm^−2^)
Carbon	0.14–0.35	3.9·10^5^	0.0169	3.3·10^−4^
c-GQDs	0.11–0.31	8.6·10^5^	0.0161	2.9·10^−4^
γ-CD-GQDs	0.10–0.30	9.3·10^5^	0.0166	3.6·10^−4^
γ-CD-GQDs-CHI	0.02–0.19	1.8·10^4^	0.0255	2.0·10^−3^

Rate limiting step: diffusion for all electrodes

Reversibility of the system: quasi-reversible for all electrodes

Firstly, nature of limiting rate step in the electrochemical process was addressed. As shown in Fig. S6, in all cases better correlation (in terms of *R*^2^ coefficient) was obtained between the oxidation current peak and the square root of the scan rate. This noticed that electrochemical process was mainly kinetically governed by the diffusion of the analyte from the core of the solution towards the electrode surface, and furthermore, the progressive modifications did not change the nature of this process in any case.

The influence exerted by the included modifiers on the reversibility of the redox process was also evaluated. For reversible processes, potential difference between anodic and cathodic peaks (*ΔE*) remains constant with scan rate variation, whereas for irreversible and quasi-reversible processes *ΔE* increases for higher scan speed values. As also displayed in Fig. S5, all attempts evidence a quasi-irreversible redox process, but however, attending the reached *ΔE* values for the sequential modifications of designed bionanocomposite, the reversibility of electronic transference was greatly improved on final γ-CD-GQDs-CHI electrode.

Kinetic constant of electronic exchange (*k*^0^) has been studied with the aim of evaluating how the designed composite affect the catalysis of the electrochemical reactions on the electrode surface. Additionally, its influence on the electroactive area (*A*) of WE electrode has also been determined. Graphical fits relative to the determination of *k*^0^ (*ψ* vs *v*^−1/2^) and *A* (*Ip* vs *v*^1/2^) are shown in Fig. S7 and S8, respectively; both parameters have been calculated from the slope of their corresponding linear fits. Regarding the obtained results, a notable increase in the electronic transfer rate is observed when introducing the c-GQDs and γ-CD-GQDs; however, the maximal value is not reached until CHI is introduced into the nanocomposite. As already explained, due to the high solubility of the GQDs, part of the nanocomposite modification resulted finally dispersed in the drop, then it was necessary to incorporate a polymer (CHI) enabling that modification stays anchored to electrode surface, but since it is a non-conductive polymer, an excess could limit the electronic transfer rate. With regard to electroactive area values, similar effects to previously described were noticed (Table 1).

Double capacitance layer (*Cdl*) was also calculated to provide information about the magnitude of capacitive component of the current. Capacitive current does not provide analytical information and sometimes may difficult the measurement of small currents associated with low analyte concentrations. Fig. S9 displays the corresponding voltammograms and graphical fits. Although an increase for the capacitive current was observed, as expected, when introducing CHI into the nanocomposite modification, it did not significantly affect the analytical determination of FQs.

As concluding remarks, modification of SPCE with γ-GQDs-CHI allows to improve sensitivity of the sensor by enhancing electronic transference between electrode surface and target solution in terms of *k*^0^ and *A*, as well as reversibility of the redox process.

### Voltammetric study of FQs and potential oxidation mechanism

**Fig. 2:**
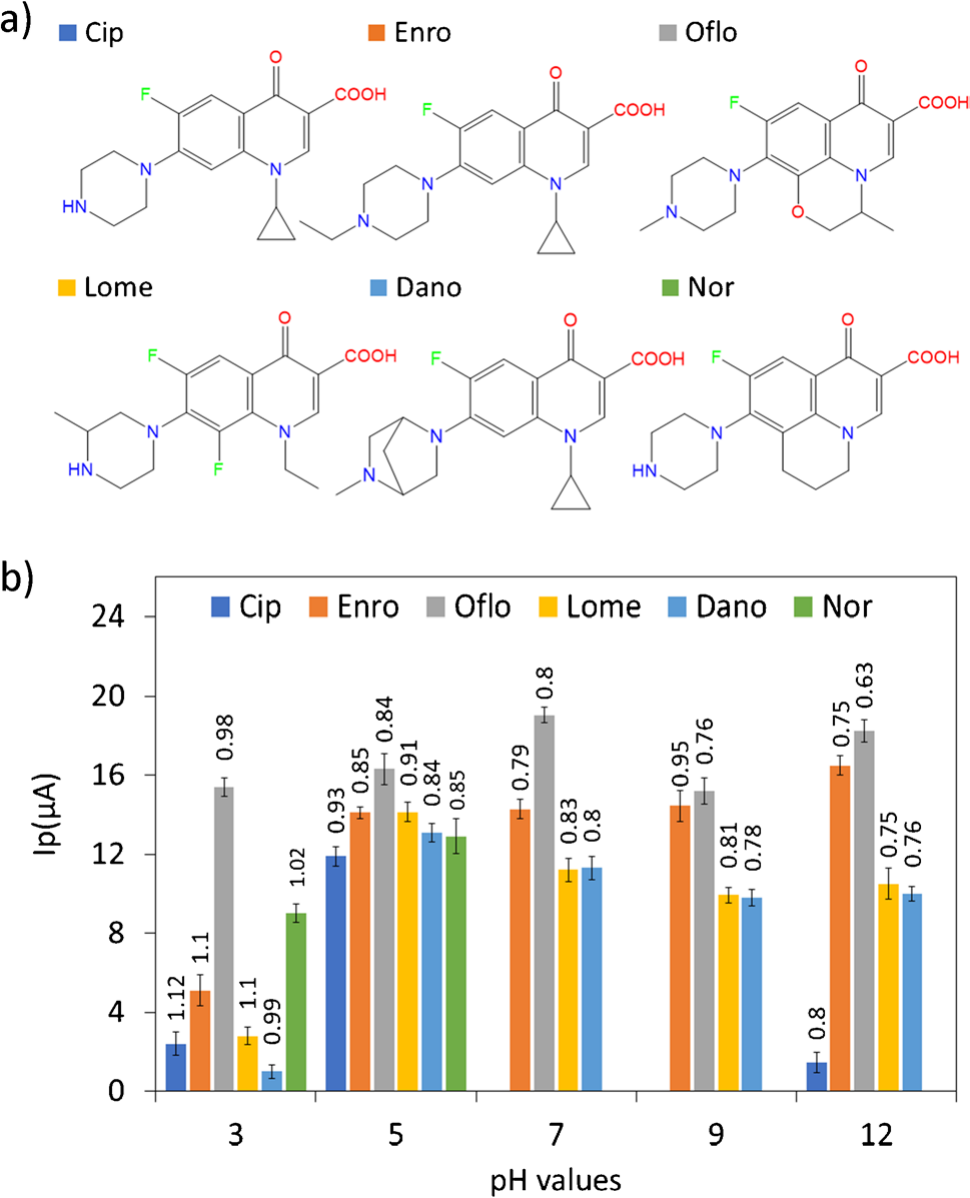
**a** Chemical structures of representative FQs (ciprofloxacin, enrofloxacin, ofloxacin, lomefloxacin, danofloxacin and norfloxacin). **b** pH influence on their peak current values. Potential peak values (volts) are displayed on the top of each bar. (*n* = 3) measurements in all cases

Firstly, the optimal working pH was evaluated since it results a critical parameter for an efficient performance of the electrochemical sensor. Thus, various FQs with heterogeneous representative substituents but having the same characteristic 1,4-dihidroquinoleine ring were checked as model analytes in this study, namely, Cip (ciprofloxacin), Enro (enrofloxacin), Nor (norfloxacin), Oflo (ofloxacin), Lome (lomefloxacin) and Dano (danofloxacin) (Fig. 2a). Individual FQs solutions (1.51 mM) were analysed by DPV between 0 and 1.2 V within a 3–12 pH range, evaluating their intensity and potential peak, *Ip* and *Ep*, for the selection of the optimal pH.

Figure 2b reveals a heterogeneous trend in the *Ip* variation for the different FQs when modifying the pH of media. Cip and Nor precipitated at basic pH values being thus these pHs discarded. At low pHs, *Ep* values of most FQs are delayed, overlapping their associated oxidation bands with that solvent oxidation and preventing their determination, therefore. Regarding the rest of results, it was observed that experiments performed at pH 5 provided appreciable and similar oxidation peaks for all studied FQs, being this value selected as the most suitable pH for the performance of the sought sensing system.

Then, at selected pH = 5, the involved species in the analyte-containing solution-modified electrode interphase are found as follows: carboxyl groups of GQDs are negatively charged (ca. *pKa* = 4) [28], amine groups of CHI are protonated (ca. *pKa* = 6.2) [29] and FQs are generally in their cationic forms, because of their *pKa* (amine group) = 7.5–8.5 and *pKa* (carboxyl group) = 5.5–6.5 [30].

**Fig. 3:**
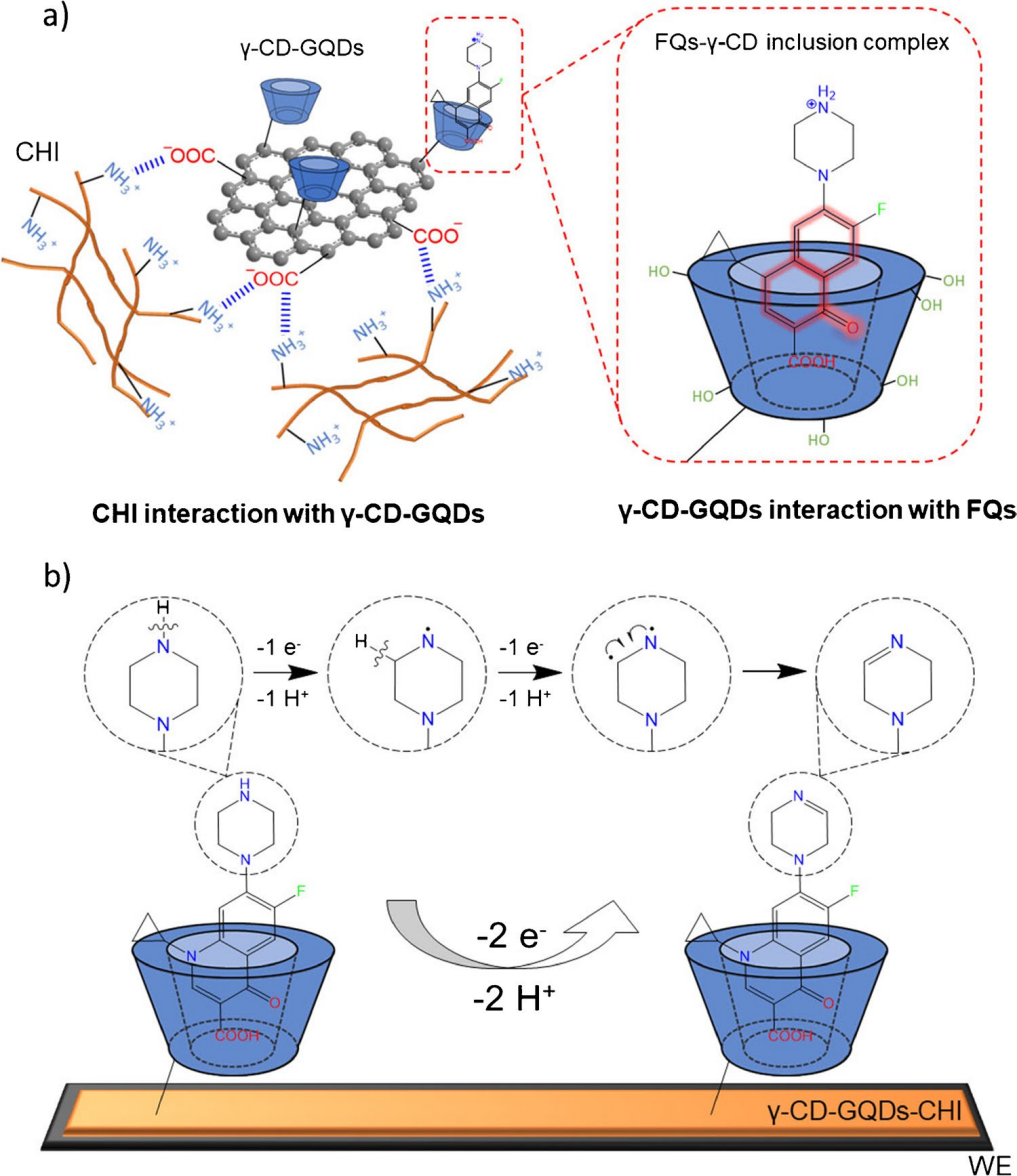
**a** Electrostatic interaction mechanism between γ-CD-GQDs and chitosan as linker polymer. (Inset) Host–guest recognition mechanism between FQs and γ-CDs to get an inclusion complex. **b** Scheme for the electrochemical oxidation mechanism of fluoroquinolones on the γ-CD-GQD-CHI/SPCE (detailly described in the second last paragraph of “Voltammetric study of FQs and potential oxidation mechanism” section)

A scheme about the anchoring of γ-CD-GQD-CHI nanocomposite to the electrode surface is drawn in Fig. 3a. Negatively charged modified GQDs are joined by electrostatic interactions between their COO^−^ functional groups and the NH_3_^+^ groups of the CHI polymer. Due to its viscosity and low solubility, CHI adheres perfectly to the electrode surface, even after several electrode washes and activation processes. In this way, CHI acts as a linker polymer, keeping the γ-CD-GQDs attached to the electrode surface.

As the inset in Fig. 3a also shows, the recognition mechanism of the sensing bionanocomposite is based on a specific host–guest interaction between γ-CDs and FQs, owing to the torus shape of the cavitand that contains a hydrophilic exterior with a hydrophobic inner wall suitable to accommodate inside the low polarity 4-oxo-1,4-dihydroquinoline ring of FQs (bright red line), which is able in turn to interact with CD inner, forming a stable inclusion complex. In this dynamic system, the driving forces enabling the host–guest interaction are the contributions from the release of enthalpy-rich water molecules from CD inner cavity besides of hydrophobic and H-bonds attending FQs structures [31].

Now, the electrochemical behaviour of FQs on the modified electrode was addressed. To elucidate a potential mechanism answering the oxidation behaviour of the whole family, four representative quinolones with different *R* substituents (Enro, Dano, Nor and Lome), but having the same characteristic 1,4-dihidroquinoleine ring were selected as analyte models.

For evaluating the number of electrons exchanged in these oxidation processes, cyclic voltammograms were performed at different scan rates (10–500 mV s^−1^) for a 1.5 mM solution of each FQ (pH = 5). The number of electrons was calculated by the Laviron equation [32].

1 $${E}_{p}={E}_{o}^{^{\prime}}+2.303\frac{RT}{\alpha nF}\mathrm{log}\;{v}_{b}-2.303\frac{RT}{\alpha nF}\mathrm{log}\left(\frac{RT\;{k}^{0}}{\alpha nF}\right)$$

where “*E*_0_^′^” is the formal potential (V), “*α*” the electronic transfer coefficient, “*k*^0^” the electronic transference constant (cm s^−1^), “*n*” is the number of involved electrons, “*v*_*b*_” the scan rate (V s^−1^), “*R*” the ideal gas constant (*R* = 8.3145 J mol^−1^ K^−1^), “*F*” the Faraday constant (*F* = 96,485.3365 C mol^−1^) and “*T*” the temperature (K). The number of involved electrons was determined by plotting the anodic peak potential (*Ep*) vs the logarithm of scan rate (log *v*_*b*_); then, from the slope value, the coefficient “*αn*” was obtained in the Laviron equation.

The electronic transfer coefficient “*α*” was calculated by Bard-Faulkner equation [33].

2 $$a=\frac{0.0477\;(V)}{(E_p-E_{p/2})}$$

where “*E*_*p*_” and “*E*_*p*/2_” are the potential and half-wave potential of anodic peak respectively expressed in volts. Since “*α*” remains practically constant and independent with the scan rate variation, an average value was taken for the “*n*” calculation of each FQ. Thus, electronic transfer coefficients calculated for Enro, Lome, Nor and Dano were 0.34, 0.33, 0.39 and 0.41, respectively.

The corresponding voltammograms for each FQ at different scan rates, as well as their linear fits for “*n*” calculations, are displayed in Fig. S10. Upon our experimental results and in accordance with other previously obtained from other researchers, 2 electrons were involved in the electrooxidation process for the four checked targets [16, 30, 34–36].

To know the number of protons (H^+^) involved in their respective oxidation process, the influence of pH on the peak potential values must be evaluated. With this aim, DPV voltammograms for 1.51 mM FQ individual solutions were registered on the bionanocomposite modified electrode within a pH range between 3 and 6. Fig. S11 displays the corresponding voltammograms and plots of anodic peak potentials vs pH values too. “H^+^” value was obtained from the slope of this linear fit upon Eq. 3:

3 $$m=0.059\;\left(\frac{H^+}n\right)$$

where “*m*” is the slope value from the *Ep* vs pH linear fit; the ratio “*H*^+^*/n*” stand for the number of protons per electron involved in this redox process, and the value “0.059” refers to the Nernstian constant in volts. Experimental *m* absolute values for the electrooxidation of the corresponding FQs were 0.0629, 0.0639, 0.0567 and 0.0579 V for Enro, Lome, Nor and Dano, respectively, that means exchange of 1 H^+^/electron. According to the obtained experimental results, there is a total exchange of 2 electrons and 2 H^+^ in the electrooxidation process for the four checked FQs. These results agree with those reported by other authors, even although other electrode modifiers were used [16, 30, 34–38].

These experimentally obtained insights (2 electrons and 2 H^+^) were considered to propose a single electrooxidation mechanism for the whole FQs family. Despite previous researchers have tried to extensively describe the electrooxidation mechanism of FQs, a broad understanding is still incomplete with some controversial aspects. Most studies (theoretical and experimental) agree that oxidation occurs on the ending nitrogen of piperazinyl ring [16, 35, 37], generating cyclic imines as final product. Based on this, a mechanistic reaction route is proposed allowing to describe a single electrooxidation behaviour for the whole FQs family for the first time, as our experimental results suggest (Fig. 3b). A brief description of the elucidated mechanism involves mainly three steps: (i) oxidation of the terminal amine group with the loss of the corresponding hydrogen atom, (ii) oxidation of the carbon atom in the position α respect to the amine group and loosing of the second hydrogen atom and (iii) rearrangement of the π electron density and formation of the imine group.

Although this mechanism is common to all FQs having a piperazine ring, there may be slight changes in the oxidation peak characteristics due to the specific structure of every molecule. In this way, the assorted substituents in the surroundings of piperazinyl ring may change the polarization in the molecular electronic density, which in turn can slightly modify the oxidation potential as well as the peak intensity and morphology [30]. Anyway, such variations related to oxidation peaks were not enough to prevent the determination of the global content of FQs as experimentally was proved by means of detailed subsequent studies for the four representative model analytes.

### Electroanalytical performance features

Analytical performance features were evaluated using the DPV peak intensity (*Ep* = ca. 0.84 V) obtained for equimolar mixtures of the four already selected representative quinolones (enrofloxacin, norfloxacin, lomefloxacin and danofloxacin) in 0.1 M acetate buffer (pH = 5), as analytical signal. Precision of voltammetric sensing approach was firstly checked by using an 80 µM FQs equimolar mixture and RSD (%) values were evaluated for both intensity (*Ip*) and potential (*Ep*) oxidation peak. For repeatability test, RSD values of 4.37% (*Ip*) and 1.42% (*Ep*) were obtained (*n* = 9, one electrode). In the reproducibility study, which was carried out at a higher level of exigency, 6.25% (*Ip*) and 1.91% (*Ep*) were obtained as RSD values (*n* = 5, three different electrodes and all solutions newly prepared). Based on these results, an excellent precision of the voltammetric bionanocomposite sensing methodology was verified considering the simplicity of electrode drop-casted modification process and the usual low reproducibility associated to SPCEs manufacturing process between different batches. Individual data for this precision study are supplied in Tables S5 and S6 of SM file.

**Fig. 4:**
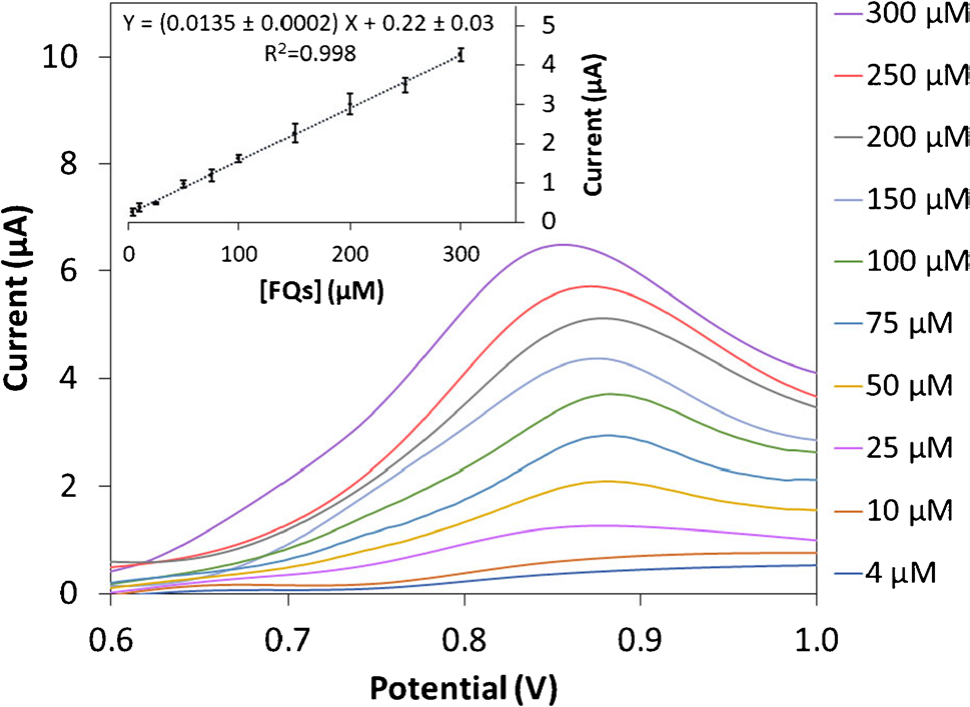
Differential pulse voltammograms and calibration plot with the confidence intervals (inset) for FQs equimolar mixtures in increasing amounts between 4 and 300 µM, at the selected γ-GQDs-CHI modified SPCE

Linear behaviour was checked for equimolar mixtures of the four selected FQs (10 levels, *n* = 3) with very good accordance within a concentration range between 4 and 300 µM. The obtained equation was *Y* = 0.0135*X* ± 0.0002 + 0.220 ± 0.003 with a regression coefficient of *R*^2^ = 0.998. The corresponding linear fit and their voltammograms are shown in Fig. 4.

LOD and LOQ values were 1.2 and 4.0 µM, respectively. LOQ was established as the lowest FQs concentration that the developed electrochemical approach was able to quantify; it means, the lowest concentration of the calibration range following indications of EURACHEM guidelines [38]. LOD was defined as LOQ/3.33 according to consulted literature [39]; its IUPAC value was displayed in Table 2.

**Table 2:** Comparative summary about the performance of current nanomaterial-based voltammetric approaches for FQs determination

Electrochemical sensor nanocomposition	Target analyte	Sensitivity (LOD) (µM)	FQs selectivity versus	Application	Reference
NiO–AgNPs/GCE	Levofloxacin	0.03	Ions, vitamins, aas, sugars and other pharmaceuticals	Human serum	[36]
Anti-quinolone antibody/SPdCEs	Enrofloxacin	0.008	Other pharmaceuticals	Meat samples	[40]
Au/PDDA/rGO/GCE	Levofloxacin	3.9	Ions, vitamins, sugars, other FQs, citric acid and urea	Pharmaceutical formulations	[41]
MWCNTs-MIP/SPCE	Norfloxacin	0.002	Ions, vitamins, other FQs, sugars, dopamine, uric acid and 5-hydroxytryptamine	Pharmaceutical formulations and rat plasma	[42]
GO/SPCE	Ciprofloxacin	0.3	Ions, vitamins and sugars	Milk	[43]
β-CD-MWCNTs/GCE	Ciprofloxacin	3.3	Ions and other pharmaceuticals	Wastewater treatment plant effluent	[44]
CNT-V_2_O_5_NPs-CHI/SPCE	Ciprofloxacin	0.0015	Other pharmaceuticals	Milk	[45]
Co-Fe-PBA/CNE-GCE	Ciprofloxacin	0.001	Ions, vitamins, aas, sugars, hormones and other pharmaceuticals	Human urine and blood serum	[46]
PVP/CPEs	Levofloxacin	10	Ions, amino acids and sugars	Pharmaceutical formulations, urine and human serum	[17]
γ-CDs-GQDs-CHI/SPCE	FQs global determination	0.8	Ions, amino acids, vitamins and sugars	Processed food (bouillons) and dairy product (milkshake)	This work

LOD values were estimated upon IUPAC criteria

Selectivity has been checked by means of an exhaustive study about the influence over the selected FQs electrochemical signal of main interferences usually present in different food products from animal origin (bouillon cubes, broths and different milkshakes). These samples exhibit complex matrices, which contain a large number of compounds that could prevent the selective determination of FQs. However, most of them can be easily removed by filtration and decantation, either because they have a lipophilic character leading to a heterogeneous dual phase system where these liposoluble species remain in the organic phase (just taken out) and then do not affect the voltammetric measurements of FQs-hydrophilic phase, or because they easily precipitate by centrifugation processes under certain conditions. Following this procedure, several potential interferences have been eliminated: some unspecific lipids and lipophilic additives not clearly specified (upon declared composition), some lipophilic vitamins such as ergocalciferol (D2), cholecalciferol (D3), γ-tocopherol and δ-tocopherol (two isomers of vit E), proteins and suspended particles of different nature.

Other potential interferences of interest remained in the aqueous phase are namely different amino acids coming from meat and milk proteins as lysine (Lys), phenylalanine (Phe), alanine (Ala), glutamic acid (Glu. A), aspartic acid (Asp. A), some hydrophilic vitamins like riboflavin (Rib), and ascorbic acid (Asc.A), ions (Na^+^, Mg^2+^, Cl^−^, SO_4_^2−^), and different carbohydrates, such as glucose (Glu) or the starch components amylose (Ams) and amylopectin (Amp). They were evaluated since they may exhibit electrochemical signals close to FQs oxidation peak upon consulted literature.

**Fig. 5:**
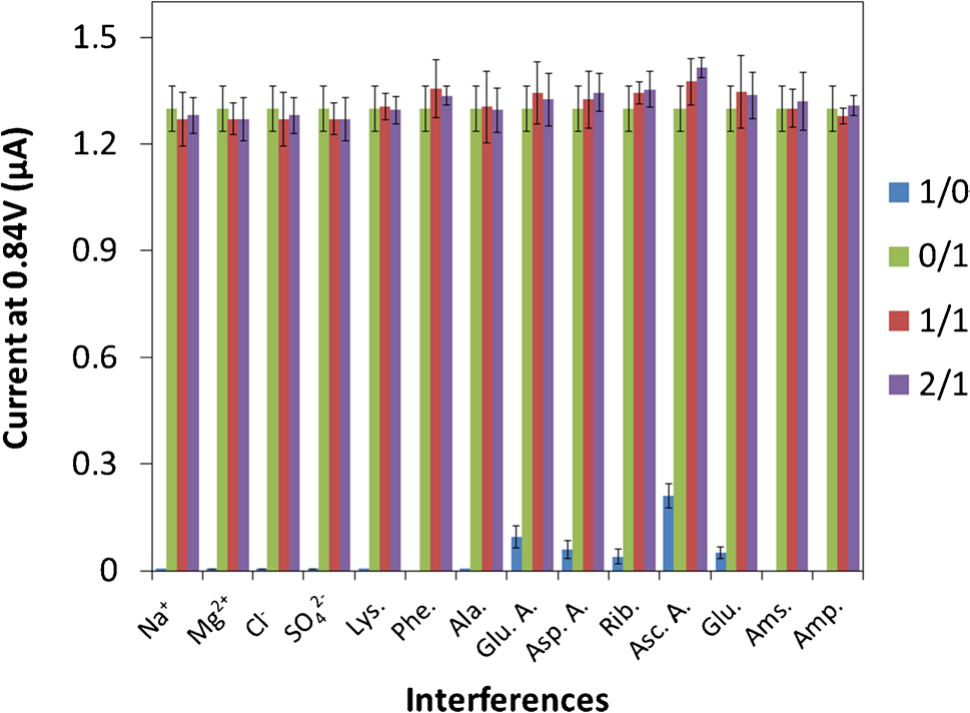
Selectivity study on the analytical signal of a 80 µM FQs equimolar mixture for the evaluation of most common interferences in commercial food samples such as amino acids, hydrophilic vitamins, ions and carbohydrates in interference/global FQs ratios: 1/0, 0/1, 1/1 and 2/1. (*n* = 3) measurements in all cases

In this specificity study, solutions containing different ratios of interference/global FQs (1/0, 0/1, 1/1, 2/1), for the before cited interferences, were prepared at pH = 5 (acetate buffer 0.1 M). The evaluation of their influence was checked on the peak intensity at 0.84 V (average potential value for the FQs equimolar mixture) of an 80 µM FQs equimolar solution, which was selected as suitable analytical signal. As Fig. 5 proves, the selected analytical signal remains practically unchanged almost in all cases either in the absence (0/1 ratio) and presence of interferences (1/0, 1/1 and 2/1 ratios), except for glutamic and ascorbic acid; apart from that, such analytical signal variations for FQs determination result lower than 10%, even in presence of these two troublesome compounds.

These results allowed us to perform reliable measurements without appreciable errors regarding the estimation of FQs global content in complex matrix, such as the sought commercial products, e.g. broths, bouillon cubes and milkshakes display.

Finally, Table 2 displays a comparison about the analytical performance reached by the developed electrochemical sensing approach regarding other recently reported nanomaterial-based methods for FQs determination. With regard to these ones, the research here proposed shows a noteworthy sensitivity and a very good selectivity in presence of a great number of common interferences (*n* = 20). But from our point of view, the main advantage of this bionanocomposite as electrochemical sensors lies on their ability to achieve the total assessment of FQs (whatever their nature or amount are) in assorted mixtures from complex samples, unlike the others which only allowed to determine a single FQ compound.

## Application to food products from animal nature

The designed bionanocomposite γ-GQDs-CHI/SPCE was applied for the voltammetric sensing of the global FQs content in five different alimentary products (chicken bouillon, veal bouillon, veal broth, chicken broth and milkshake strawberry flavour) upon the pre-treatment procedures already described in “Experimental” section (Sample treatments). All samples were spiked at three different FQs concentration levels (37.5, 75 and 150 µM) and submitted to the electrochemical procedure already detailed in that section. All performed analyses were carried out by fivefold.

Firstly, the accuracy of the electrochemical sensing approach for the analysis of complex matrix was checked by spiking the five commercial samples with equimolar FQs solutions at the three concentration levels. Table 3 displays the recovery values, as well as their corresponding RSD. Excellent agreement was reached between added and found amounts in all cases (recoveries ranged from 89.6 to 106.1%) despite the expected variability inherent to the use of disposable and home-made drop-casted modified electrodes. Although the interferences study has proved this sensing approach as selective enough for the determination of FQs, and due to the complexity of the target matrix, it was necessary to dilute certain samples for carrying out the analysis to avoid excessive WE surface fouling.

**Table 3:** γ-CD-GQDs-CHI/SPCE voltammetric analysis of the global FQs amount in commercial food samples spiked at three concentration levels (37.5, 75, 150 µM)

Food sample	Initial spiked amount (µM)	Matrix dilution	Found (µM)	% recovery (*n* = 5)	% RSD (*n* = 5)
Chicken bouillon cube	150	1:2	152.5	101.6	8.7
	75	1:2	79.6	106.1	8.1
	37.5	1:2	39.7	105.9	2.4
Veal bouillon cube	150	1:4	145.5	97.0	9.0
	75	1:4	79.4	105.8	8.5
	37.5	1:4	39.0	104.1	5.8
Chicken broth	150	1:8	135.2	90.2	9.8
	75	1:2	74.4	99.1	11.3
	37.5	1:2	35.0	93.4	7.6
Veal broth	150	1:8	158.7	105.8	10.5
	75	1:4	69.0	92.0	12.0
	37.5	1:4	38.4	102.2	8.2
Milkshake strawberry flavour	150	1:4	151.8	101.2	1.3
	75	1:4	67.6	90.2	10.4
	37.5	1:4	33.6	89.6	2.4

Furthermore, to verify the reliability about the predictive ability of γ-CD-GQDs-CHI/SPCE approach for the voltammetric sensing of FQs mixtures with assorted composition and ratios in a more realistic scenario, five new designed experiments (one for each product) were carried out again under the same conditions, but with variable content and presence of the four involved quinolones. Global and specific amounts of each FQ as well as the obtained recoveries (*n* = 5) are specified in Table 4, where values between 89.2 and 106.3% are reached in all cases. This fact proves the excellent predictive ability of developed sensing approach, whatever was the structure or relative amounts of any involved FQ specie.

**Table 4:** γ-CD-GQDs-CHI/SPCE voltammetric analysis of global FQs amount in commercial food samples spiked with variable ratios of the four representative FQs

Food sample	Initial spiked amount (µM)	E/L/N/D^*^ ratios	Found (µM)	% recovery (*n* = 5)	% RSD (*n* = 5)
Chicken bouillon cube	37.5	(0/2/1/1)	33.4	89.2	6.2
Veal bouillon cube	150	(1/0/1/0)	143.0	95.4	7.5
Chicken broth	75	(0/1/0/0)	68.4	91.0	8.2
Veal broth	150	(1/1/0/2)	159.2	106.3	8.5
Milkshake strawberry flavour	150	(1/3/1/3)	135.6	90.4	2.9

^*^E enrofloxacin, *L* lomefloxacin, *N* norfloxacin, *D* danofloxacin

Besides, the accuracy of the recovery results obtained in this section was successfully evidenced by statistical comparison tests. With this aim, the determination of the global FQs amount on synthetic samples prepared at the same concentration levels as the commercial ones was carried out by fivefold, following the indications of the “Statistics and Chemometrics for Analytical Chemistry Manual” by Miller and Miller [47]. All calculations relative to these statistical comparison tests as well as the involved results are detailly described in section S4 of SM file, as well as in their corresponding Tables S2-S4.

Finally, considering the dilution factors applied to the commercial samples, the determination of the FQs global content was carried out at a minimal concentration around 1500 µg (FQs)/kg (sample), but attending the estimated LOQ, quantification of FQs could be carried out at levels up to 400 µg (FQs)/kg (sample). Thus, upon the legislation established by European health organizations [6], where the maximal FQs residues are limited between 30 and 800 µg (FQs)/kg (sample), the developed sensing approach can become a useful quality control tool for the screening of FQs residues in potential contaminated food, with the aim to fight against antimicrobial resistance issues.

## Conclusions

A novel engineered electrochemical bionanocomposite γ-CD-GQDs-CHI modified SPCEs has been revealed for the full voltammetric sensing of FQs in alimentary products. Physic-chemical, nanostructural and electrochemical features have been submitted to an extensive optimization research and characterizations. It has been evidenced an improved electrochemical behaviour towards oxidation of fluoroquinolones (LOD = 1.2 µM) as well as about reusability issues (*n* = 10) due to excellent conductivity of GQDs incorporated into chitosan film and because of size based γ-CD discrimination for selective FQs recognition as versatile recognition tools over a large number of interferent compounds (almost twenty) usually present in processed products suspicious of FQs residues.

Based on the experimental behaviour and electrochemical insights (number of exchanged electrons and protons), it has been also elucidated for the first time a single oxidation mechanism for the whole FQs family, attending to chemical structures of representative selected targets, in opposite to previous works where such study was just addressed to single species.

It has been proved the applicability of the designed bionanocomposite/SPCE approach since it has provided high performance analysis of FQs mixtures in assorted composition and ratios. Despite the existence of some electrochemical methodologies for FQs detection, all of them just provided sensing of individual or up to two quinolone species simultaneously, almost the same ones, ciprofloxacin or enrofloxacin. Thus, the submitted approach constitutes a turning point in the development of a versatile and low-cost methodologies for the control of these family drugs in food matrices suspicious of such contamination, thus establishing a valuable tool in the fight against antimicrobial resistance issues whose seriousness is currently growing exponentially.

Besides, the developed methodology focusses its application on complex and processed foods as broths, bouillon cubes and milkshakes are. Most of SPCE sensors so far designed for FQs determination are just applied to some specific matrices such as milk or eggs, which limit the versatility of the analysis or even to very simple samples such as water ones which are not representative about the troublesome and realistic scenario where the determination of FQs should be considered.

Notwithstanding, two main limitations related to reusability of sensor were found, firstly, the loss of modified layer between measurements due to the high water-solubility of GQDs which were solved by the inclusion of CHI as linker polymer in an optimized percentage to avoid excessive sensitivity decrease and, secondly, the loss of current due to fouling of WE itself, resolved by the renewing of the electrode surface every two measurements, although as setoff, this anodic oxidation process would extend the useful life of the modified SPCE up to 10 measurements.

## Supplementary Information

Below is the link to the electronic supplementary material.

Supplementary file1 (DOCX 2717 KB)

## Data Availability

The data presented in this study are available on request to the corresponding author.
