# Supplementary material for: γ-Cyclodextrin-graphene quantum dots-chitosan modified screen-printed electrode for sensing of fluoroquinolones
Source: Mikrochim Acta. 2023 Jan 19;190(2):60. doi: 10.1007/s00604-023-05646-w (PMC9852125; doi:10.1007/s00604-023-05646-w)
Supplement: Supplementary file 1 — Supplementary file1 (DOCX 2717 KB) [file 604_2023_5646_MOESM1_ESM.docx]

**Electronic Supplementary Material**

**ɣ-cyclodextrin-graphene quantum dots-chitosan modified SPCE for sensing of fluoroquinolones**

**Manuel Bartolomé^1,2^, M. Laura Soriano^1,3^, M. Jesús Villaseñor^1.4^, Ángel Ríos*^1,2^**

*^1^Department of Analytical Chemistry and Food Technology, Faculty of Chemical Science and Technology, University of Castilla-La Mancha, 13071, Ciudad Real, Spain*

*^2^Regional Institute for Applied Chemistry Research (IRICA), 13071, Ciudad Real, Spain*

*^3^Department of Analytical Chemistry, University of Córdoba, Campus of Rabanales, Marie Curie, E-14071, Córdoba, Spain; qa2sodom@uco.es*

*^4^Department of Analytical Chemistry, Industrial Engineering School, University of Castilla-La Mancha, 13071, Ciudad Real, Spain*

**EXPERIMENTAL**

**S1.** Synthesis of c-GQDs, α-CD-GQDs, β-CD-GQDs, γ-CD-GQDs and γ-CD-GCDs/CHI modifiers

**S2.** Theoretical background parameters for electrochemical characterization

**RESULTS AND DISCUSSION**

**S3.** Structural, morphological and optical characterization of c-GQDs, α-, β- and γ-CD-GQDs

**S4**. Accuracy comparative statistical tests of recovery results

**FIGURES**

**S1.** A) Scheme for preparation of functionalized GQDs with γ-CDs. B-C) Monographs obtained for the γ-GQDs by HR-TEM

**S2.** Hydrodynamic diameter distributions of c-GQDs, α-, β- and γ-CD-GQDs

**S3.** XRD and FTIR characterization of c-GQDs, α-, β- and γ-CD-GQDs

**S4.** Optical (UV-Vis and fluorescence) characterization of c-GQDs, α-, β- and γ-CD-GQDs

**S5.** Cyclic voltammograms of potassium ferricyanide (5 mM) in KCl (0.1 mM) on bare and modified electrodes at scan rates ranging from 10 to 500 mV s^-1^ and their recorded ΔE.

**S6.** Study about absorption/diffusion-controlled limiting rate step on bare and modified electrodes.

**S7.** Graphical depictions for kinetic constant rate calculations on bare and modified electrodes

**S8.** Graphical depictions for electroactive areas calculations on bare and modified electrodes

**S9.** Graphical depictions for capacitance layer on bare and modified electrodes

**S10.** Determination of the number of electrons involved in the oxidation process for the four selected FQs: a) Enro b) Lome c) Nor and d) Dano

**S11.**  Determination of the number of protons involved in the oxidation of the four selected FQs: a) Enro b) Lome c) Nor and d) Dano

**TABLES**

**S1.** Maximal emission and excitation wavelengths and FWHM for c-GQDs, α-, β- and γ-CD-GQDs

**S2.** Data obtained for standards analysis

**S3.** Data obtained for commercial samples spiked with equimolar FQs mixtures and statistical comparison with standards

**S4.** Data obtained for commercial samples spiked with FQs mixtures in assorted composition and ratios and statistical comparison with standards

**S5.** Individual data for repeatability study.

**S6.** Individual data for reproducibility study.

**EXPERIMENTAL**

***S1. Synthesis of c-GQDs, α-CD-GQDs, β-CD-GQDs, γ-CD-GQDs and γ-CD-GCDs/CHI modifiers***

Carboxylated-GQDs (c-GQDs) were prepared following a previous method [1] with slight modifications. Uric acid (1 g) was refluxed in 2 mL sulfuric acid (18 M) for 1 h and the resulting brown solution was then neutralized and diluted up to ca. 20 mL. Then, the addition of methanol (10 mL) and the subsequent refrigeration of the resulting solution for 10 min at 5ºC lead to precipitation of carbon aggregates and salt excess. The supernatant containing c-GQDs was then treated with *N,N′-*diisopropylcarbodiimide (0.25 mmol) for 1 h to activate the oxygen-containing groups. Then, the yellowish solution was subjected to a N_2_ flow for 10 min. After filtration with a 0.2-µm pore size nylon membrane, the final solution was diluted up to a concentration of 9.6 g L^-1^ at which their photoluminescence was stable and well-dispersed for months. The functionalization with cyclodextrins occurs through a double esterification process in which succinic acid behaves as the bridge between c-GQD edges (with activated carboxylic groups) and cyclodextrins. First, a solution containing succinic acid (25 mL of 5.55 µM, as crosslinking agent) was mixed in a 100-mL round-bottom flask with 10 µL of 43 µM [Ru(bpy)_2_(a-bpy)](PF_6_)_2_ catalyst solution, synthesized upon ref. [2]. Second, with medium stirring, 10 mL of previously activated GQDs (9.6 g L^-1^) was added maintaining pH 5, and third, addition of 10 mL 0.5 g L^-1^ γ-CDs was performed under strong-stirring dropwise conditions for 2 min to reach 13.9 µM as final concentration of the cavitand. The solution was then allowed to react for 24 hours, protecting it from light radiation. Later, γ-CD-GQDs solution was filtered and preconcentrated in a rotavapor (60ºC, 14000 rpm) to reach a 5 mL final volume and it was subsequently dialyzed in dialysis tubing-bags (35 kDa cut-off size) for 2 h. Finally, the fluorescent final product was diluted with deionized water up to 10 mL and storage in darkness until use.

Synthesis procedures for GQDs functionalized with α-CDs and β-CDs were the same as the already described for γ-CDs. Final concentrations of 4.5, 4.5 and 4.1 g L^-1^ for α-CD-GQDs, β-CD-GQDs and γ-CD-GQDs were respectively obtained.

***S2. Theoretical background parameters for electrochemical characterization***

The potential difference (*ΔE_p_*) for a redox couple on each modified electrode surface is given by the following equation:

$${{\Delta E}_{p}=Ep}_{c}-{Ep}_{a} (1)$$

where Ep_c_ and Ep_a_ are the cathodic and anodic peak potentials, respectively. This electrochemical parameter provides information about the reversibility trend for a redox process, since smaller values lead faster electron kinetics; in this way, for a whole reversible process *ΔEp* is not dependent on scan rate (*v*), while for irreversible processes, a linear dependence exists between these two variables.

Nature of limiting rate step (diffusion or adsorption controlled) can be evaluated through the linear relationship between the peak intensity (*Ip*) and the applied scan rate (adsorption) or between *Ip* and the square root of scan rate (diffusion).

The electron transfer rate constant (*k^0^*) provides quantitative information about the rate at which electronic exchange occurs. This parameter was obtained by means of Nicholson equation (equation 2)[3].

$$Ѱ=k^{0}{(D_{O}/D_{R})}^{\alpha/2}{(\pi nFDv)}^{-1/2}{(RT)}^{1/2} (2)$$

where *D_0_* and *D_r_* are the diffusion coefficients of the oxidized and reduced species, respectively (cm^2^ s^-1^). ***α*** is the transfer coefficient, ***n*** the number of exchanged electrons (*n*=1 for Fe(CN)_6_^3-^/Fe(CN)_6_^4-^ redox pair), ***F*** is the Faraday constant (F=96485.3365 C mol^-1^), ***v*** the scan rate (V s^-1^), ***R*** is the universal gas constant (R=8.314 J mol^-1^ K^-1^) and ***T*** the temperature (K). Considering that (*D_O_ ≈ D_R_*), simplified Nicholson equation resulted to be:

$$Ѱ=k^{0}{(\pi nFDv)}^{-1/2}{(RT)}^{1/2} (3)$$

To determinate *k^0^*, dimensionless function (*Ψ*) was first obtained for each scan rate according to Swaddle equation (4), using *ΔEp* as its respective value for a specific scan rate [4]:

$$Ln Ѱ=3,69-1,16\cdot Ln\left( \Delta E_{p}-59 \right) (4)$$

After that, *k^0^* was obtained from the slope value in the plot of dimensionless function versus square root of scan rate.

The electroactive areas (*A*) of the bare and the different modified WEs were calculated following the Randles-Sevcik equation (5), [5]:

$$I_{p}={2,69\cdot10}^{5}\cdot C\cdot{A\cdot D}^{1/2}\cdot n^{3/2}\cdot v^{1/2} (5)$$

where ***I_p_*** is the peak intensity (A), ***C*** is the analyte concentration (mol cm^-3^), ***A*** is the electroactive area (cm^2^) and ***D*** is the diffusion coefficient of electroactive species (D = 0.726 ± 0.011·10^-5^ cm^2^ s^-1^)[6]. Value of electroactive areas were achieved from the slope of linear plots corresponding to peak intensity versus the square root of scan rate.

Capacitance layer of the electrode-solution interface (***Cdl***) was calculated according to Eq. (6):

$$I_{c}=A \cdot Cdl \cdot v (6)$$

where ***I_c_*** is the capacitive current (µA) and ***Cdl*** is the capacitance of the double layer (µF cm^-1^). This value was obtained from the obtained slope after plotting capacitive current versus scan rate.

**RESULTS AND DISCUSSION**

***S3. Structural and morphological characterization of*** ***c-GQDs, α-CD-GQDs, β-CD-GQDs and γ-CD-GQDs***

In this research, c-GQDs were surface-functionalized with cyclodextrin moieties using succinic acid as the bridging agent, in contrast to other works employing ethylenediamine as the linker [7]. **Fig. S1A** schematically displays the synthesis of functionalized GQDs with γ-CDs, protocol also used for preparing GQDs decorated with the other two types of cavitands, α-CDs and β-CDs.

Spherical-like nanodots with 3.8–4.7 nm as diameter were found, with graphitic morphology and with a mean hydrodynamic size of 25.2 nm (n=5), as examined by HR-TEM and DLS respectively (**Fig. S1B** and **Fig**. **S2**). **Fig. S1C** shows a graphitic lattice structure with an interplanar distance of 0.33 nm typical of the (002) facet of graphitic carbons [8]. As expected, the hydrodynamic diameter of the functionalized GQDs is larger than that c-GQDs and gradually increases with the cyclodextrin size (**Fig. S2**). In contrast to well-dispersed and stable dispersions obtained for all of them, the Zeta potential values for the modified GQDs decreases with larger cyclodextrin diameter, being -6,03, -9,58, -11,9 and -18,5 mV for the α-CD-GQDs, β-CD-GQDs, γ-CD-GQDs and c-GQDs, respectively, likely due to the number of available surface carboxylic groups. This fact may be explained attending to the reduced number of cavitands linked to the GQDs carboxyl ending groups, with the lowest one for γ-CDs due to its steric hindrance.

XRD pattern of the γ-cyclodextrin modified GQDs, **(Fig. S3A**) exhibits a broad peak centered at 26° associated with the (002) graphite plane, which is consistent with the highly disorder graphene-like structures observed for similar nanomaterials in other works [9].

The functional modification of GQDs was assessed by FT-IR spectroscopy. **Fig. S3B** shows FTIR spectra corresponding to GQDs modified with α-, β- and *γ*-CDs while **Fig. S3C** reveals the typical IR peaks of c-GQDs, raw γ-CDs and the modified γ-CD-GQDs. Interestingly, a vibration band located at ca. 1734 cm^− 1^ (**Fig. S3B**) was noticed for the three cyclodextrin-modified GQDs and assigned to a C=O stretching vibration mode typical of ester bonding.

All three CDs modified GQDs displayed similar excitation and emission properties than the c-GQDs as depicted in **Fig. S4** (A-D). A comparison of the fluorescence emission in GQDs with carboxylic groups or functionalized with α-, β- or γ-cyclodextrins in aqueous phase at neutral pH corroborates that the PL emission is invariable with change in excitation energy for any of them. Herein, it is confirmed how cyclodextrins (used as recognition element) contribute to the broadening of the PL emission of GQDs as a result of the mid-gap states formed from the deformation of the aromatic core, which is consistent to other report [10]. The contributions of both blue and red shoulders in the reported GQDs spectra are higher as the cyclodextrin moiety increases in size. Thus, GQDs functionalized with γ-cyclodextrins in aqueous phase at neutral pH display the highest FWHM (full width at half maximum), followed by β- and α-CD-GQDs (**Table S1**). Absorption spectra of γ-CD-GQDs (Fig. S3E) exhibited two maximum absorbance at 257 and 364 nm, being the first one attributed to the π → π* transition of the aromatic sp^2^ domains and the latter due to the carbene-like states and n-π* transition.

***S4. Accuracy comparative statistical tests of recovery results***

The reliability of the proposed method was proved by means of an exhaustive validation procedure where the accuracy of the recovery results was evidenced by statistical comparison tests. So, with this aim, the determination of the global FQs amount on synthetic samples prepared at the same concentration levels as the commercial samples were carried out by fivefold. Results obtained for synthetic samples (**Table S2**) were statistically compared with those ones from commercial samples (**Tables S3** and **S4**) following the indications of the “**Statistics and Chemometrics for Analytical Chemistry Manual”** by Miller [11].

At first, Fisher test was performed in order to corroborate the statistical homogeneity of variances between results obtained in standards and commercial samples. Statistical hypotheses were raised ($H_{0}: \sigma_{1}^{2}=\sigma_{2}^{2}$ and $H_{1}: \sigma_{1}^{2}\neq\sigma_{2}^{2}$) and the values ​​of F (Equation 1) and F _(a/2; n1-1; n2-2)_ were calculated (Table 2 and 3).

$$F=S_{1}^{2}/S_{2}^{2} (1)$$

Where $S_{1}^{2}$ and $S_{2}^{2}$are the squares of the standard deviation of standards and commercial samples results respectively, (n_1_-1 and n_2_-2) are the number of freedom degrees of the numerator and denominator.

As shown in **Tables** **S3** and **S4**, experimental F values were in all cases lower than the critical F value _(0.025;4;3)_, therefore alternative hypothesis was rejected, and it can be assessed that the variance of the results obtained by both series does not show significant differences.

Once the homogeneity of the variances between the results has been proved, the paired Student test-t was performed between these two sets of data to verify the statistical accordance between results reported by both series. Null and alternative hypothesis were established as $H_{0}: \mu_{1}=\mu_{2}$and $H_{1}: \mu_{1}\neq\mu_{2}$; t_0_ (Equation 2) and t _(α/2;n-1)_ were calculated. The obtained values are shown in **Tables** **S3** and **S4**.

$$t_{0}=\frac{(\bar{x}_{1}-\bar{x}_{2})}{s\sqrt{\frac{1}{n_{1}}}+\frac{1}{n_{2}}} (2)$$

Were $\bar{x}_{1},\bar{x}_{2}$ are the average values and n_1_, n_2_, are the number of freedom degrees of standards and commercial samples results respectively. In order to determinate the Student t coefficient, combined standard deviation "s" was first estimated upon equation 3.

$$s= \frac{{(n}_{1}-1)s_{1}^{2}+{(n}_{1}-1)s_{2}^{2}}{(n_{1}+n_{2}-2)} (3)$$

According to the results showed at **Tables** **S3** and **S4**, in all cases t_0_ experimental values were lower than the critical one (t _(0.025;4)_); therefore, it can be guaranteed the absence of statistically significant differences between the results obtained by both series at a confidence level of 95%, which would prove their comparative accuracy at that checked confidence level.

**REFERENCES**

1. Durán GM, Benavidez TE, Contento AM, Ríos A, García CD (2017) Analysis of penicillamine using Cu-modified graphene quantum dots synthesized from uric acid as single precursor. J Pharm Anal 7(5):324-331. <https://doi.org/10.1016/j.jpha.2017.07.002>
2. Ruiz-Palomero C, Soriano ML, Valcárcel M (2016) Gels based on nanocellulose with photosensitive ruthenium bipyridine moieties as sensors for silver nanoparticles in real samples. Sens Actuators B: Chem 229:31-37. <https://doi.org/10.1016/j.snb.2016.01.098>
3. Nicholson RS (1965) Theory and application of cyclic voltammetry for measurement of electrode reaction kinetics. Anal Chem 37(11):1351-1355. <https://doi.org/10.1021/ac60230a016>
4. Swaddle TW (2005) Homogeneous versus heterogeneous self-exchange electron transfer reactions of metal complexes: Insights from pressure effects. Chem Rev 105(6):2573-2608. <https://doi.org/10.1021/cr030727g>
5. Randviir EP, Brownson DA, Metters JP, Kadara RO, and Banks CE (2014) The fabrication, characterisation and electrochemical investigation of screen-printed graphene electrodes. Phys Chem Chem Phys 16(10):4598-4611. <https://doi.org/10.1039/C3CP55435J>
6. Konopka S, McDuffie B (1970) Diffusion coefficients of ferri-and ferrocyanide ions in aqueous media, using twin-electrode thin-layer electrochemistry. Anal Chem 42(14):1741-1746. <https://doi.org/10.1021/ac50160a042>
7. Pinilla-Peñalver E, Soriano ML, Contento AM, Ríos A (2021) Cyclodextrin-modified graphene quantum dots as a novel additive for the selective separation of bioactive compounds by capillary electrophoresis. Mikrochim Acta 188(12):1-12. <https://doi.org/10.1007/s00604-021-05098-0>
8. Wu ZL, Gao MX, Wang TT, Wan XY, Zheng LL, Huang CZ (2014) A general quantitative pH sensor developed with dicyandiamide N-doped high quantum yield graphene quantum dots. Nanoscale 6(7):3868-3874. <https://doi.or/10.1039/C3NR06353D>
9. Boonta W, Talodthaisong C, Sattayaporn S, Chaicham C, Chaicham A, Sahasithiwat S, Kangkaew L, Kulchat S (2020) The synthesis of nitrogen and sulfur co-doped graphene quantum dots for fluorescence detection of cobalt (II) ions in water. Mater Chem Front 4(2):507-516. <https://doi.or/10.1039/C9QM00587K>
10. Wang S, Cole IS, Zhao D, and Li Q (2016) The dual roles of functional groups in the photoluminescence of graphene quantum dots. Nanoscale 8(14). https://doi.org/7449-7458. <https://doi.or/10.1039/C5NR07042B>
11. J. Miller and J. C. Miller, Statistics and chemometrics for analytical chemistry. Pearson education, 4^th^ Ed, 2002.

**FIGURES**


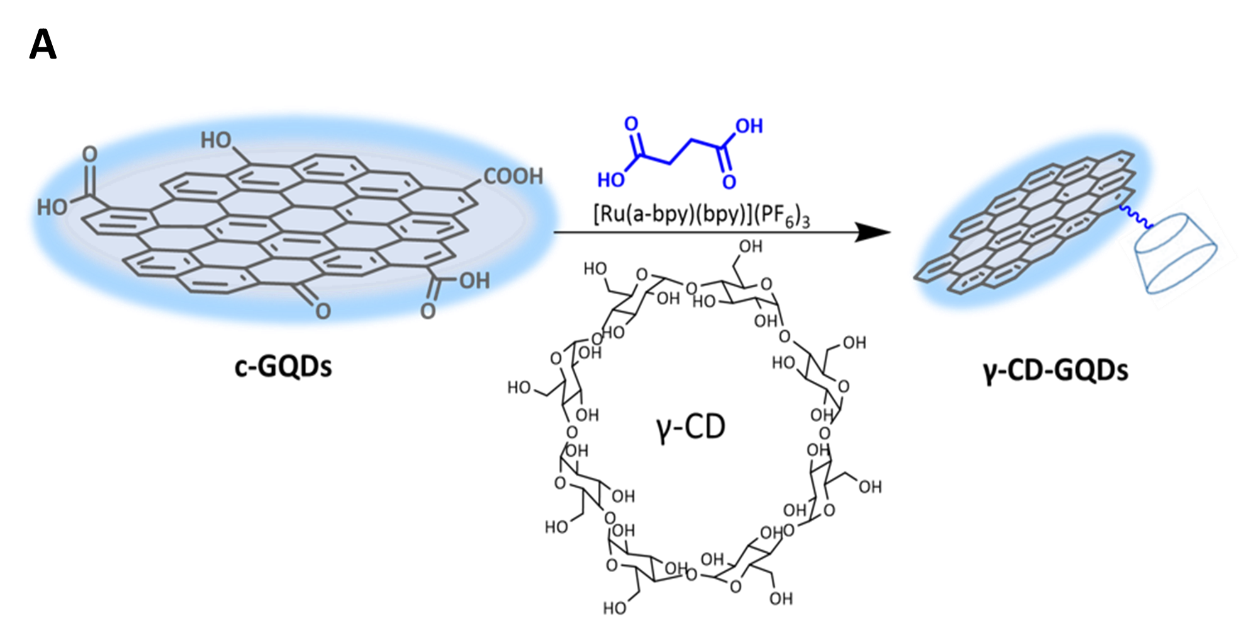


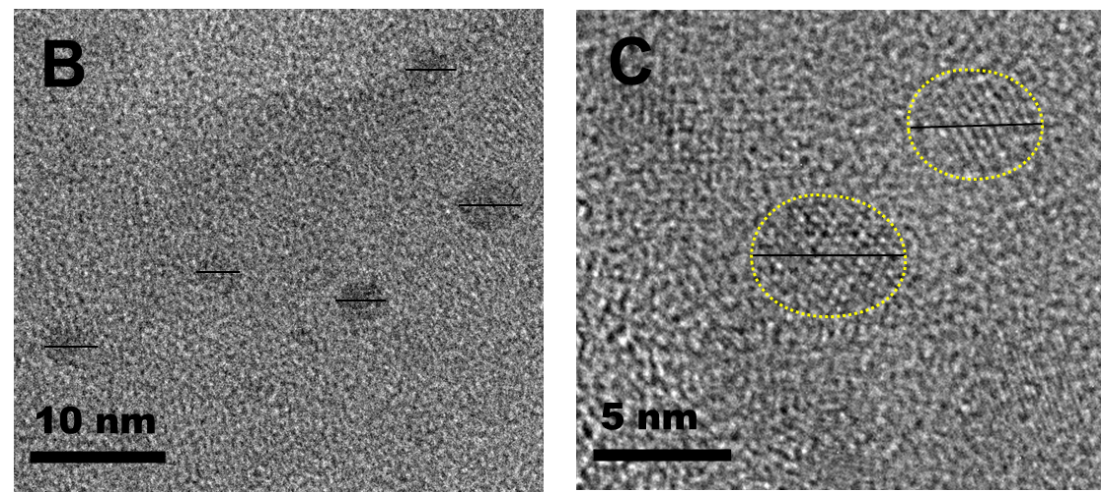


***Fig. S1*** *A) Schematic procedure for the preparation of functionalized GQDs with γ-CDs. B-C) Monographs obtained for the γ-GQDs by HR-TEM*


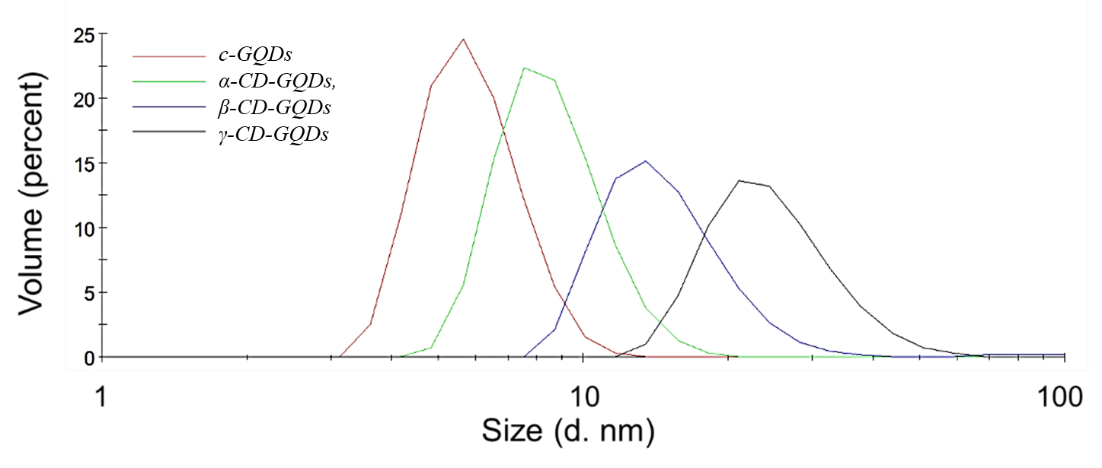


***Fig. S2*** *Hydrodynamic diameter distributions of the c**-GQDs, α-CD-GQDs, β-CD-GQDs and γ-CD-GQDs by dynamic light scattering (average diameters: 5.92, 8.67, 14.95 and 25.20 nm, respectively).*
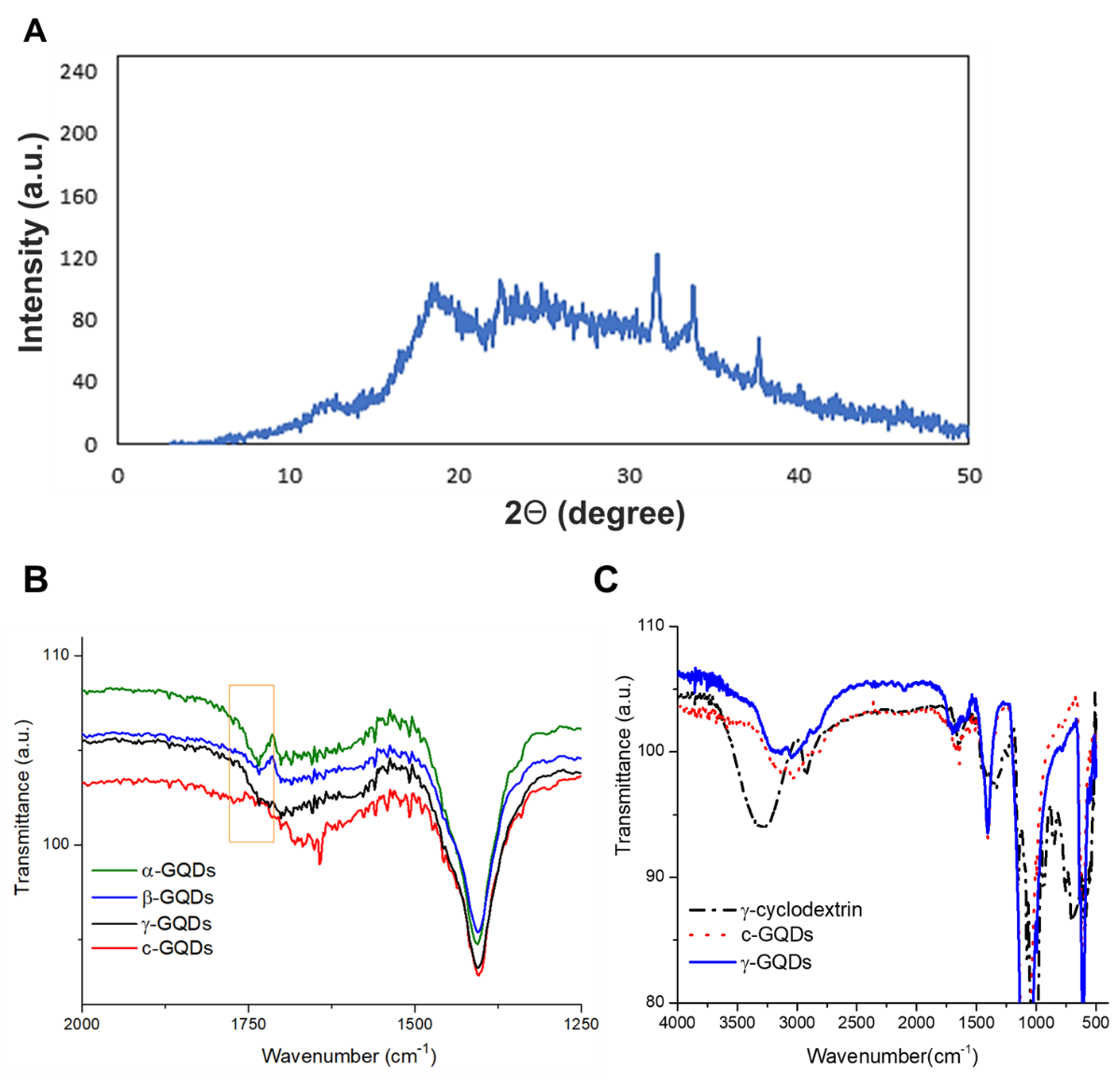


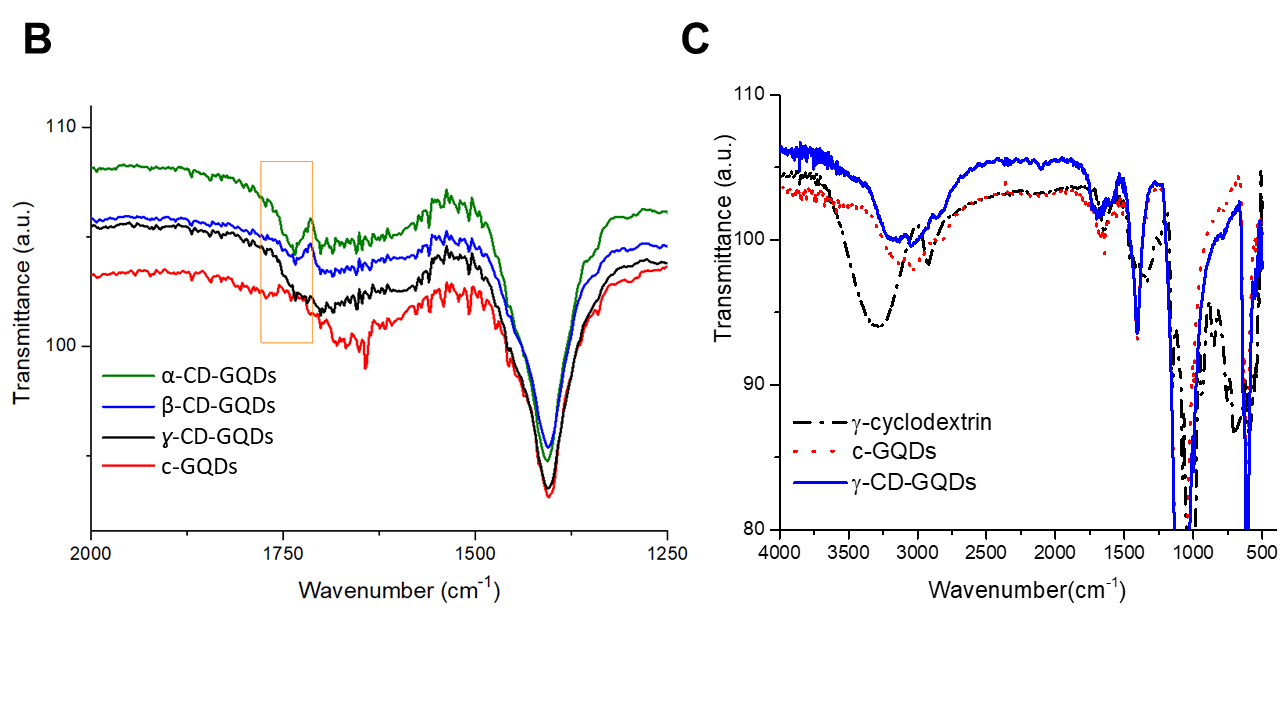


***Fig. S3*** *A) XRD spectra of the γ-cyclodextrin modified GQDs. B) FTIR spectra of raw and functionalized GQDs with the three cyclodextrins (α-, β- and γ-CD-GQDs), highlighting the new peak attributed to C═O stretching (ester formation for the coupling between cyclodextrins and the GQD surface activated carboxylic functions through the hydroxyl groups). C) FTIR spectra of γ-cyclodextrin (black dash-dot line), the carboxylated GQDs (red dotted line) and γ-cyclodextrin modified GQDs (blue solid line).*


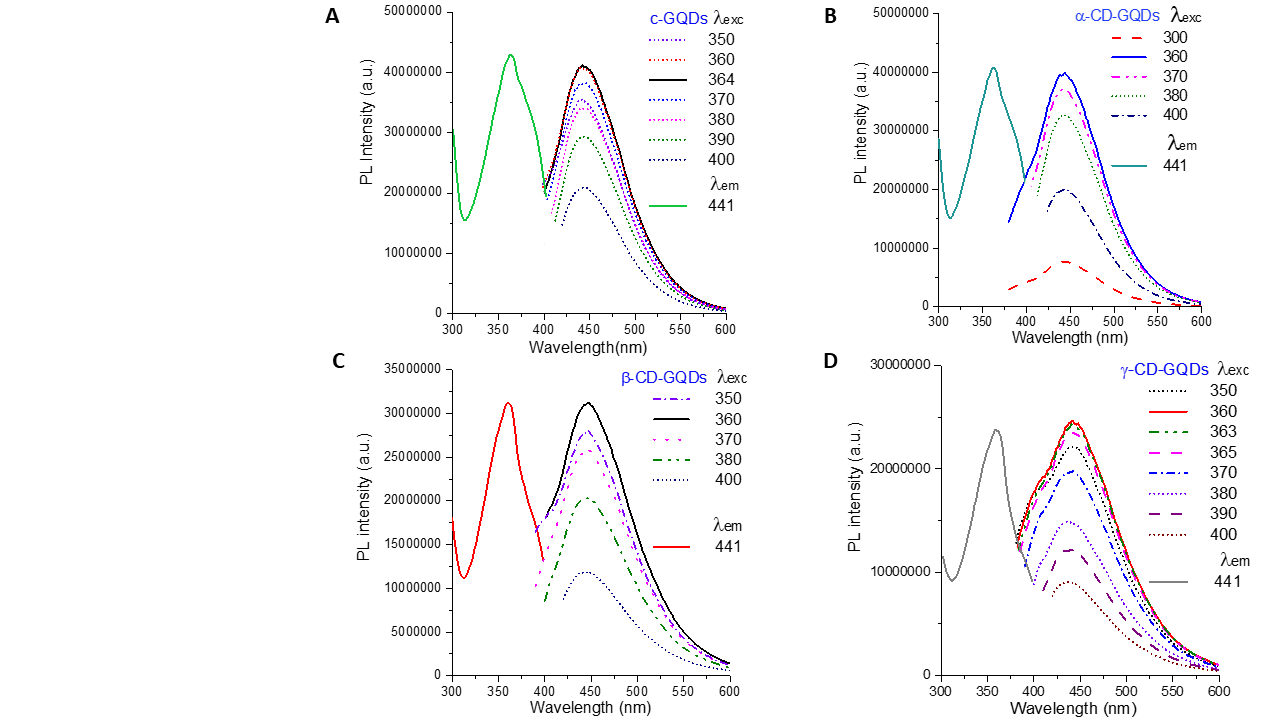


**E**


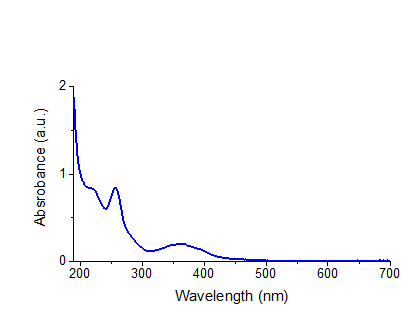


***Fig. S4*** *Excitation spectra at the maximal emission, and emission spectra at diverse excitation wavelengths of the carboxylated (A) and functionalized GQDs with α-cyclodextrin (B), β-cyclodextrin (C) and γ-cyclodextrin (D). (E) UV-Vis absorption spectra of γ-CD-GQDs*


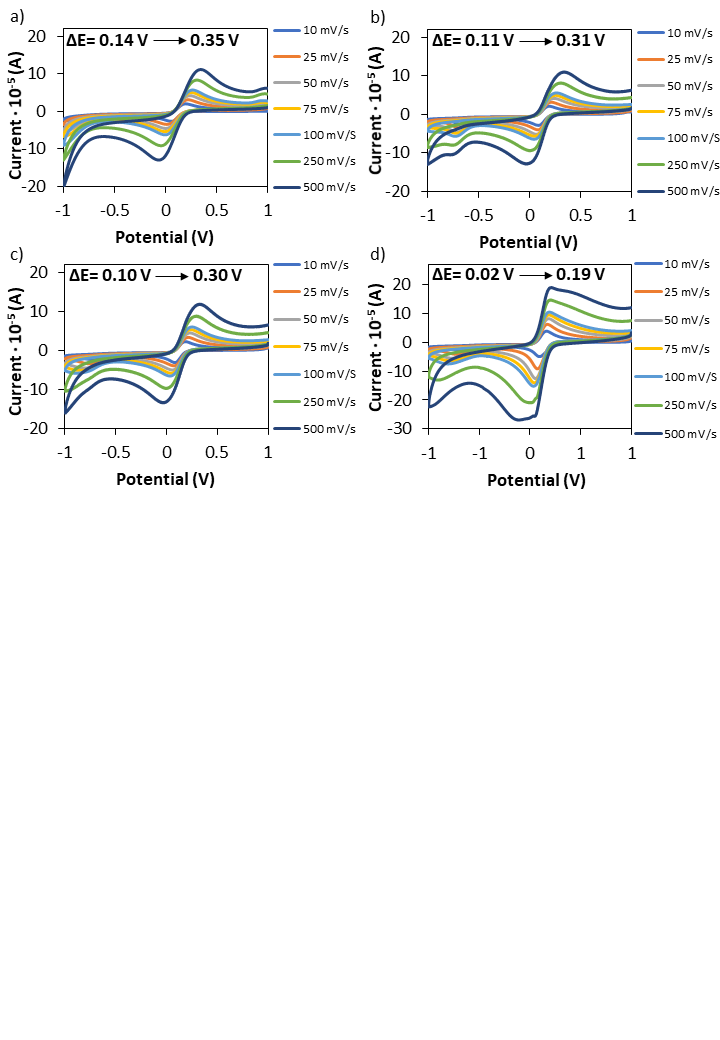


**Fig. S5** Cyclic voltammograms of potassium ferricyanide (5 mM) in KCl (0.1 mM) for a) bare SPCE, b) c-GQDs/SPCE, c) γ-CD-GQDs/SPCE, and d) γ-CD-GQDs-CHI/SPCE. Potential difference intervals obtained for scan rates ranging from 10 to 500 mV s^-1^ are also reported in the corresponding images.


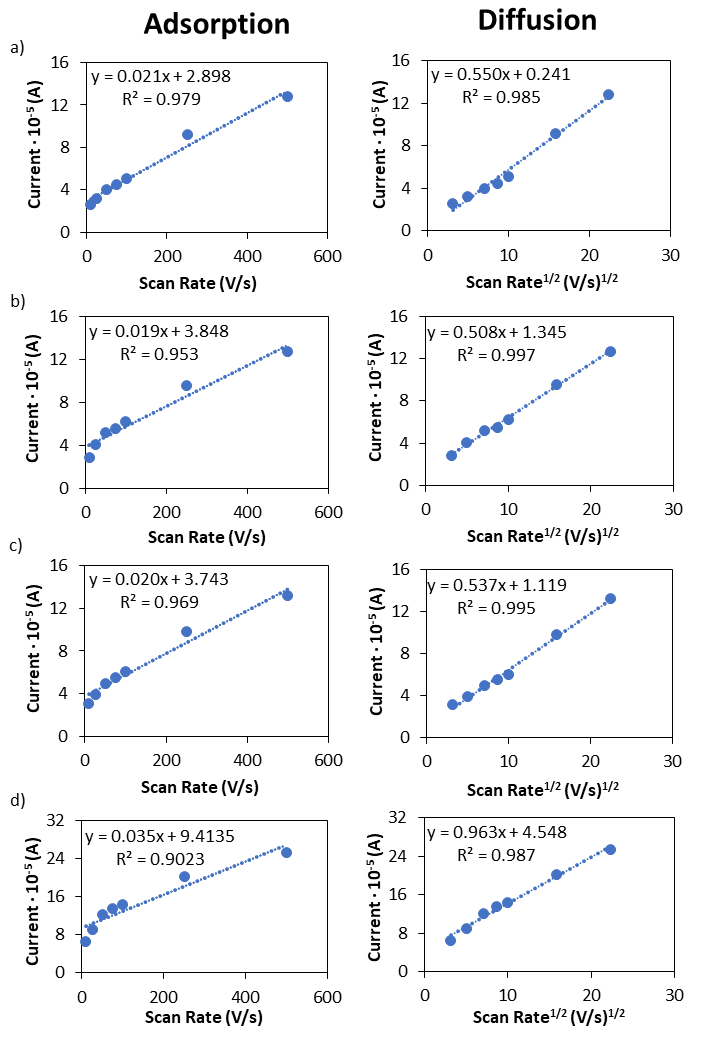


**Fig. S6** Study about the nature of limiting rate step for electrochemical process in terms of adsorption (relationship between current and scan rate) and diffusion (relationship between current and square root of scan rate) for a) bare, b) c-GQD, c) γ-CD-GQDs and d) γ-CD-GQDs-CHI modified SPCEs.


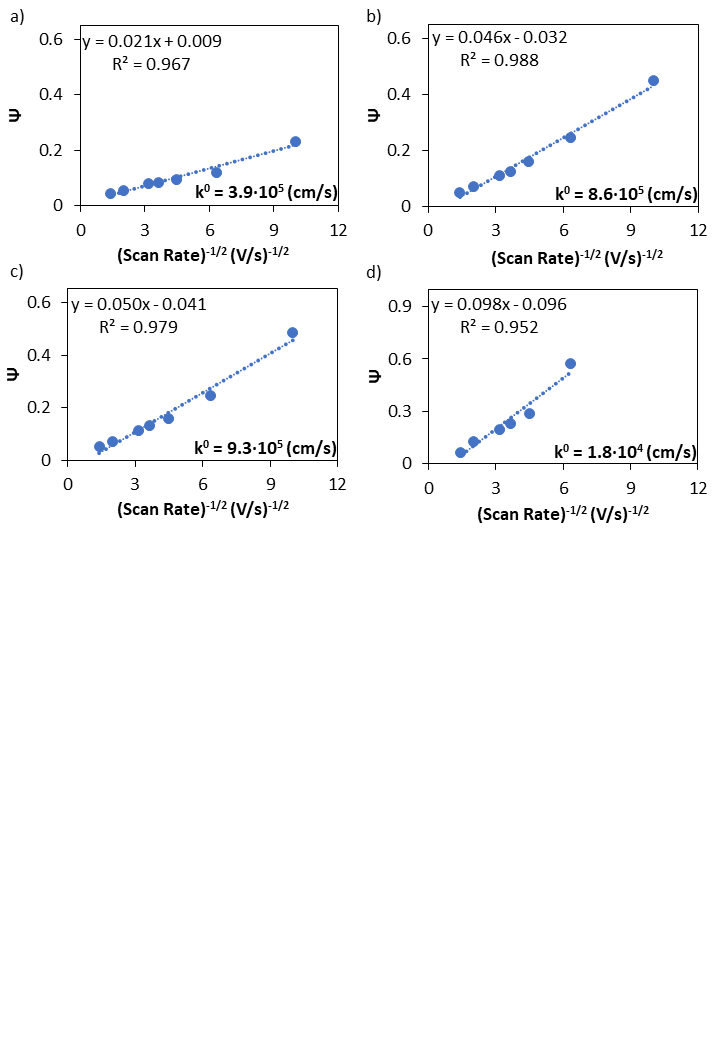


**Fig. S7** Relationship between dimensionless function and the inverse of scan rate square root for determination of kinetic constant rate at a) bare, b) c-GQD, c) γ-CD-GQDs, and d) γ-CD-GQDs-CHI modified SPCEs.


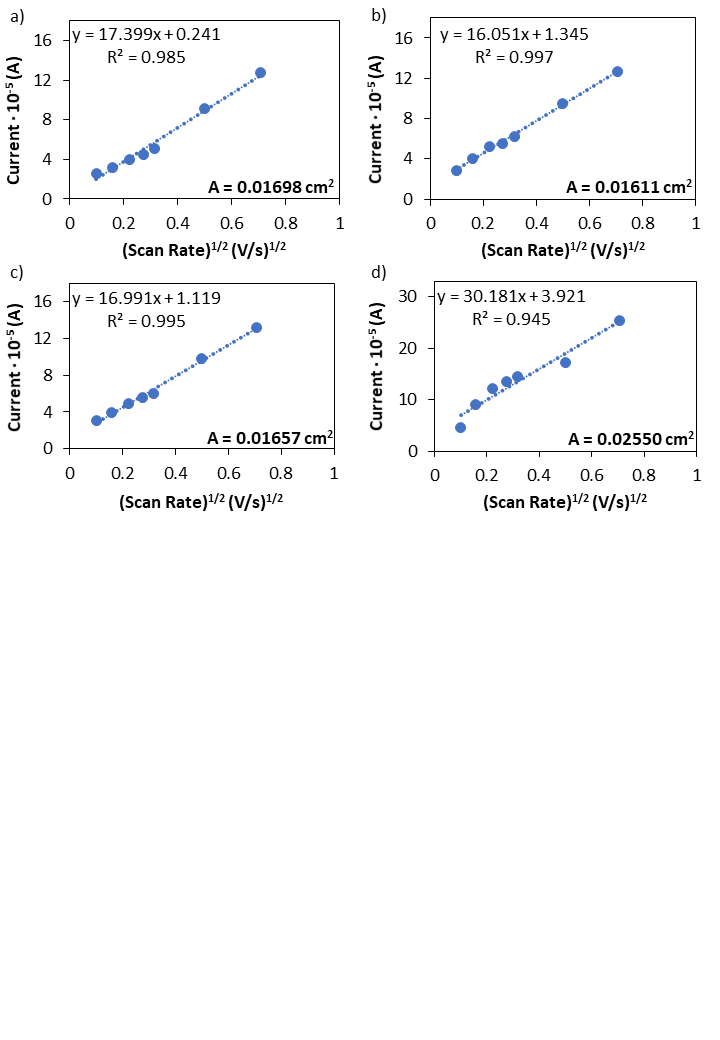


**Fig. S8** Relationship between oxidation current peak and square root of scan rate for determination of electroactive areas at a) bare, b) c-GQD, c) γ-CD-GQDs and d) γ-CD-GQDs-CHI modified SPCEs.


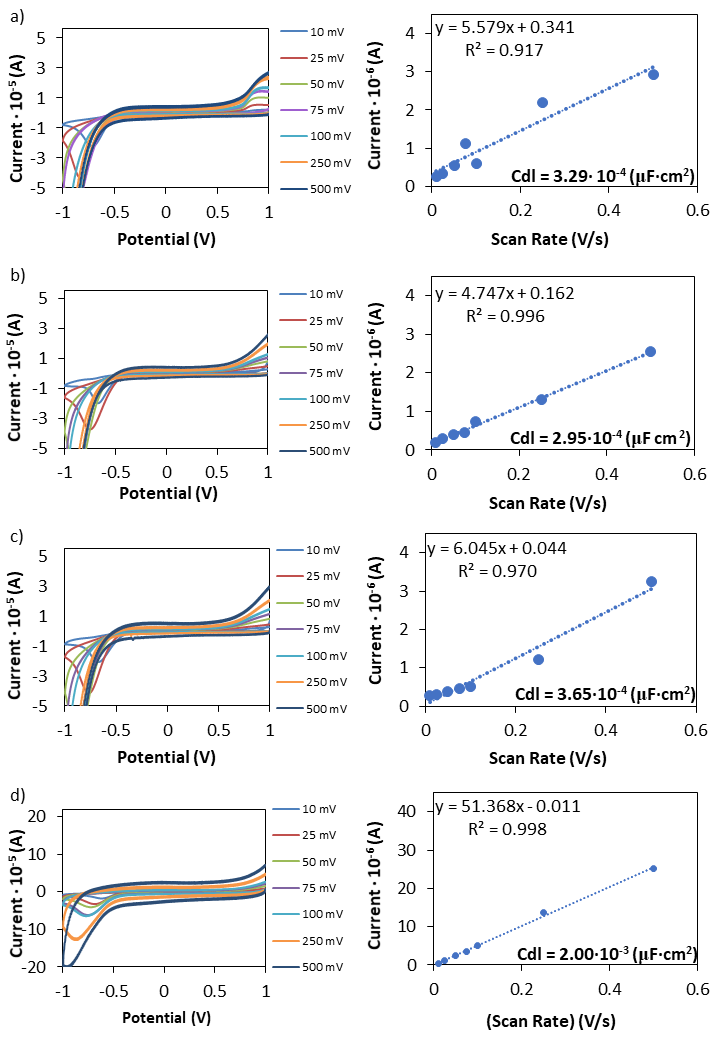
**Fig Fig. S9** Cyclic voltammograms of KCl (0.1 M) and relationship between current and scan rate for the determination of capacitance laye for a) bare, b) c-GQD, c) γ-CD-GQDs and d) γ-CD-GQDs-CHI modified SPCEs.


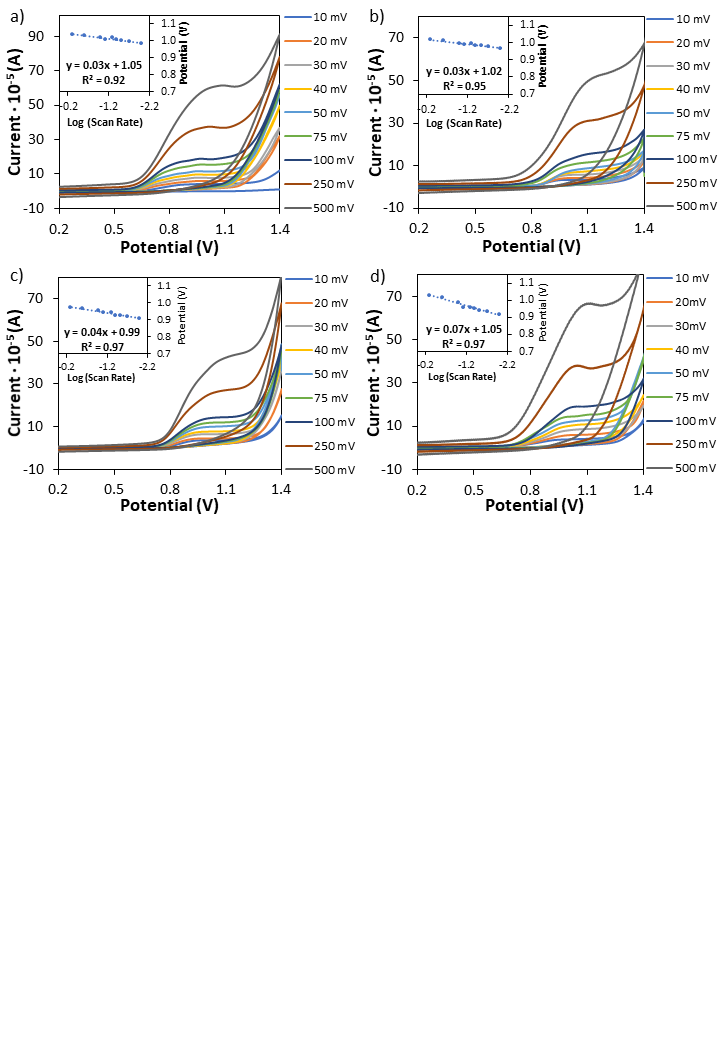


**Fig. S10** Cyclic voltammograms for the determination of the number of electrons involved in the oxidation process and their corresponding linear fits for the four selected FQs: a) Enro b) Lome c) Nor and d) Dano.


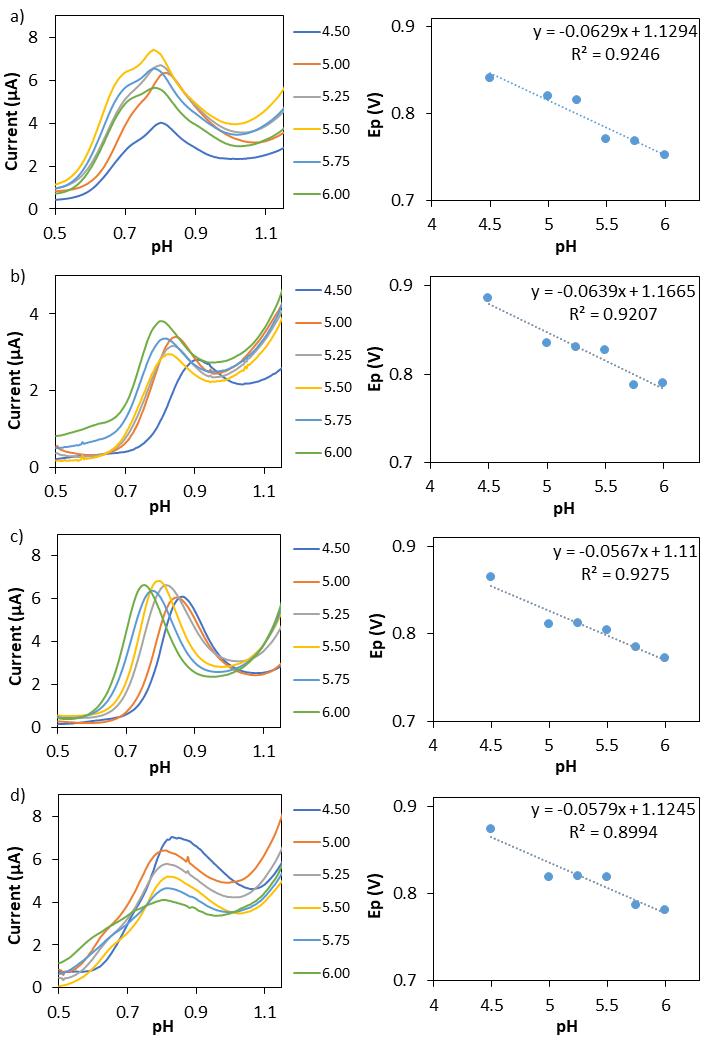


**Fig. S11** Differential pulse voltammograms for the determination of the number of protons involved in the oxidation process and their corresponding linear fits of the four selected FQs: a) Enro b) Lome c) Nor and d) Dano.

**TABLES**

***Table S1.*** *Maximal emission and excitation wavelengths and full width at half maximum (FWHM)*

*for the ensuing GQDs.*

| **Nanoparticle type** | **Excitation wavelength (nm)** | **Emission wavelength (nm)** | **FWHM (nm)** |
| --- | --- | --- | --- |
| c-GQDs | 360 | 442 | 92 |
| α-CD-GQDs | 360 | 444 | 99 |
| β-CD-GQDs | 360 | 448 | 108 |
| ɣ-CD-GQDs | 360 | 442 | 116 |

***Table S2.*** *Data obtained for standards analysis*

| Standards data | | | |
| --- | --- | --- | --- |
| Standard | Spiked amount (µM) | Found (µM)  $\bar{x}_{1}$ | Standard deviation (µM) (σ) |
| 1 | 150 | 147.9 | 5.3 |
| 2 | 75 | 73.2 | 2.3 |
| 3 | 37.5 | 36.4 | 1.9 |

***Table S3.*** *Data obtained for commercial samples spiked with equimolar FQs mixtures and statistical comparison with standards*

| Sample data | | | | Fischer test data | | Student test data | | |
| --- | --- | --- | --- | --- | --- | --- | --- | --- |
| Food sample | Initial spiked amount (µM) | Average found concentration (µM)  ($\bar{x}_{2}$) | Standard deviation (µM) (σ) | F_0_ | F_(0.025,4,3)_ | s | t_0_ | t_(0.025,4,4)_ |
| Chicken bouillon cube | 150 | 152.5 | 13.3 | 6.2 | 15.1 | 102.2 | 0.07 | 2.31 |
|  | 75 | 79.6 | 6.4 | 7.6 |  | 23.5 | 0.43 |  |
|  | 37.5 | 39.7 | 1.0 | 4.1 |  | 2.31 | 2.25 |  |
| Veal bouillon cube | 150 | 145.5 | 13.1 | 6.0 |  | 99.9 | 0.03 |  |
|  | 75 | 79.4 | 6.7 | 8.3 |  | 25.5 | 0.38 |  |
|  | 37.5 | 39 | 2.3 | 1.3 |  | 4.4 | 0.93 |  |
| Chicken broth | 150 | 135.2 | 13.2 | 6.2 |  | 101.9 | 0.20 |  |
|  | 75 | 74.4 | 8.4 | 12.9 |  | 38.1 | 0.05 |  |
|  | 37.5 | 35 | 2.7 | 1.9 |  | 5.4 | 0.41 |  |
| Veal broth | 150 | 158.7 | 16.7 | 9.8 |  | 153.0 | 0.11 |  |
|  | 75 | 69 | 8.3 | 12.5 |  | 37.0 | 0.18 |  |
|  | 37.5 | 38.4 | 3.1 | 2.7 |  | 6.8 | 0.46 |  |
| Milkshake strawberry flavour | 150 | 151.8 | 2.0 | 7.3 |  | 16.1 | 0.38 |  |
|  | 75 | 67.6 | 7.0 | 9.0 |  | 27.4 | 0.32 |  |
|  | 37.5 | 33.6 | 0.8 | 5.7 |  | 2.2 | 2.03 |  |

***Table S4.*** *Data obtained for commercial samples spiked with FQs mixtures in assorted composition and ratios and statistical comparison with standards*

| Sample data | | | | | Fischer test data | | Student test data | | |
| --- | --- | --- | --- | --- | --- | --- | --- | --- | --- |
| Food sample | Initial spiked amount (µM) | E/L/N/D^*^ | Average found concentration (µM)  ($\bar{x}$) | Standard deviation (µM) (σ) | F_0_ | F_(0.025,4,3)_ | s | t_0_ | t_(0.025,4,4)_ |
| Chicken bouillon cube | 37.5 | (0/2/1/1) | 33.4 | 2.1 | 1.2 | 15.1 | 4.0 | 1.18 | 2.31 |
| Veal bouillon cube | 150 | (1/0/1/0) | 143 | 10.7 | 4.1 |  | 71.7 | 0.11 |  |
| Chicken broth | 75 | (0/1/0/0) | 68.4 | 5.6 | 5.7 |  | 18.5 | 0.41 |  |
| Veal broth | 150 | (1/1/0/2) | 159.2 | 13.5 | 6.5 |  | 105.7 | 0.17 |  |
| Milkshake strawberry flavour | 150 | (1/3/1/3) | 135.6 | 3.9 | 4.2 |  | 21.9 | 0.89 |  |

***Table S5****. Individual data for repeatability study.*

| Repeatability (n=9) | | |
| --- | --- | --- |
| Measurements | Ip (µA) | Ep (V) |
| 1 | 1.31 | 0.86 |
| 2 | 1.33 | 0.88 |
| 3 | 1.22 | 0.86 |
| 4 | 1.38 | 0.87 |
| 5 | 1.34 | 0.84 |
| 6 | 1.31 | 0.86 |
| 7 | 1.32 | 0.86 |
| 8 | 1.35 | 0.86 |
| 9 | 1.23 | 0.86 |

*%RSD ( in terms of Ip)=4.4% *%RSD ( in terms of Ep)=1.4%

***Table S6.*** *Individual data for reproducibility study.*

| Reproducibility (n_(total)_=16) | | | |
| --- | --- | --- | --- |
| Modified SPCE | Measurements | Ip (µA) | Ep(V) |
| 1 (n=5) | 1 | 1.31 | 0.86 |
|  | 2 | 1.33 | 0.88 |
|  | 3 | 1.22 | 0.86 |
|  | 4 | 1.23 | 0.86 |
|  | 5 | 1.34 | 0.85 |
| 2 (n=6) | 1 | 1.12 | 0.84 |
|  | 2 | 1.33 | 0.85 |
|  | 3 | 1.16 | 0.82 |
|  | 4 | 1.27 | 0.84 |
|  | 5 | 1.26 | 0.86 |
|  | 6 | 1.24 | 0.86 |
| 3 (n=5) | 1 | 1.19 | 0.83 |
|  | 2 | 1.12 | 0.83 |
|  | 3 | 1.15 | 0.85 |
|  | 4 | 1.31 | 0.84 |
|  | 5 | 1.33 | 0.87 |

*%RSD( in terms of Ip)=6.3% *%RSD( in terms of Ep)=1.9%
